# Role of non-coding RNA-regulated ferroptosis in colorectal cancer

**DOI:** 10.1038/s41420-025-02606-6

**Published:** 2025-07-08

**Authors:** Yan-Peng Zhao, Jun-Liang Liu, Shuai Wang, Xue Li

**Affiliations:** Department of General Surgery, Central Hospital Affiliated to Shenyang Medical College, Shenyang, China

**Keywords:** Colon cancer, Cell death, Non-coding RNAs

## Abstract

The recently discovered type of programmed cell death, termed ferroptosis, characterized by an iron-dependent accumulation of lipid peroxides, has been demonstrated to play a pivotal role in the progress of tumors. The role of non-coding RNAs (ncRNAs) in various malignant tumors has also been gradually elucidated in recent years. Colorectal cancer (CRC) is a malignant tumor with a high prevalence and mortality rate worldwide. Many recent studies have demonstrated that the effects of ncRNAs on CRC progression may be mediated by their regulation of ferroptosis. This review first outlines the fundamental mechanisms of ferroptosis and the role of ncRNAs in ferroptosis, and then we summarize the role of ferroptosis in CRC. We then focused on summarizing the effect of ncRNAs regulating ferroptosis in CRC and the recent progress of ferroptosis-related ncRNAs as prognostic biomarkers in CRC patients. This review will help to deepen our understanding of the ncRNA-ferroptosis-CRC axis and inform the study of potential therapeutic targets and prognostic markers for CRC patients.

## Facts

- Ferroptosis is a common form of cell death that can be regulated by ncRNAs.
- Ferroptosis has been shown to play an important role in the progression of various cancers, including CRC.
- NcRNAs can affect the biological behaviors of CRC such as proliferation, metastasis, and drug resistance by regulating ferroptosis.
- Ferroptosis-associated ncRNAs may serve as biomarkers for CRC to predict patient prognosis and treatment sensitivity.

## Open questions

- The role of ncRNAs-regulated ferroptosis in CRC is not yet fully understood.
- What are the specific mechanisms by which ncRNAs regulate ferroptosis in CRC?
- Is there crosstalk between ferroptosis regulated by ncRNAs and other forms of cell death in cancer?

## Introduction

Cancer is the second highest cause of death, with approximately 20 million new cases of cancer reported in 2020 worldwide, and colorectal cancer (CRC) is a common and highly malignant tumor, ranking 3rd and 2nd in terms of incidence and mortality, respectively [1, 2]. Due to the lack of effective screening and diagnostic tools, many CRC patients are already in advanced stages when they are first diagnosed, so the prognosis for CRC patients is inferior, with a five-year survival rate of not even 10% [3, 4]. With the development of medicine, great strides have been made in the diagnosis and treatment of CRC patients, but the prognosis of CRC patients is still not very good due to the emergence of recurrence, metastasis, and drug resistance [5]. Therefore, the search for new CRC biomarkers and a more in-depth study of the pathogenesis of CRC are necessary.

Non-coding RNAs (ncRNAs) include long noncoding RNAs (lncRNAs), microRNAs (miRNAs), circular RNAs (circRNAs), transfer RNAs, and ribosomal RNAs, which are ncRNAs that are not directly involved in the regulation of genetic information but still play important roles in human diseases [6–8]. Mechanistically, miRNAs can affect the translation of mRNAs by targeting them, whereas both lncRNAs and circRNAs can act as sponges for miRNAs to regulate miRNAs-mediated regulation of genetic information; moreover, ncRNAs can also affect protein function and downstream signaling pathways by directly binding to proteins [9]. In summary, although ncRNAs are not directly involved in the expression of genetic information, their role in human diseases has received widespread attention, especially in tumor diseases, and more and more evidence suggested that ncRNAs play vital regulatory roles in various tumors, and ncRNAs have become biomarkers and therapeutic targets for tumor diseases [10–13].

Ferroptosis, a new concept introduced by Dixon in 2012, differs from necrosis, apoptosis, autophagy, and other forms of cell death [14, 15], which has distinctive cellular morphology, ultrastructural, and metabolic features [16–19]. Ferroptosis is connected to a variety of human diseases, especially tumor diseases, and ferroptosis plays an essential role in tumor development, diagnosis, and treatment [20]. In addition, therapies such as chemotherapy, radiotherapy, and immunotherapy for tumors can exert tumor-killing effects by inducing ferroptosis [21–23]. Thus, ferroptosis has enormous research value in the diagnosis and treatment of tumors, and ferroptosis has emerged as a potential therapeutic target for tumors.

NcRNAs are important regulators of ferroptosis, which can affect the malignant behaviors of tumor cells such as survival, proliferation, metastasis, and drug resistance by regulating ferroptosis [24, 25].

However, the role of ncRNAs in CRC ferroptosis remains to be clarified. In this review, we first concluded the key mechanisms of ferroptosis. We then briefly summarized the relationship between ncRNAs and ferroptosis and the role of ferroptosis in CRC. Finally, we summarize the role of ncRNAs-regulated ferroptosis in CRC and the potential of ncRNAs as biomarkers for CRC patients.

## The core mechanism of ferroptosis

**Fig. 1:**
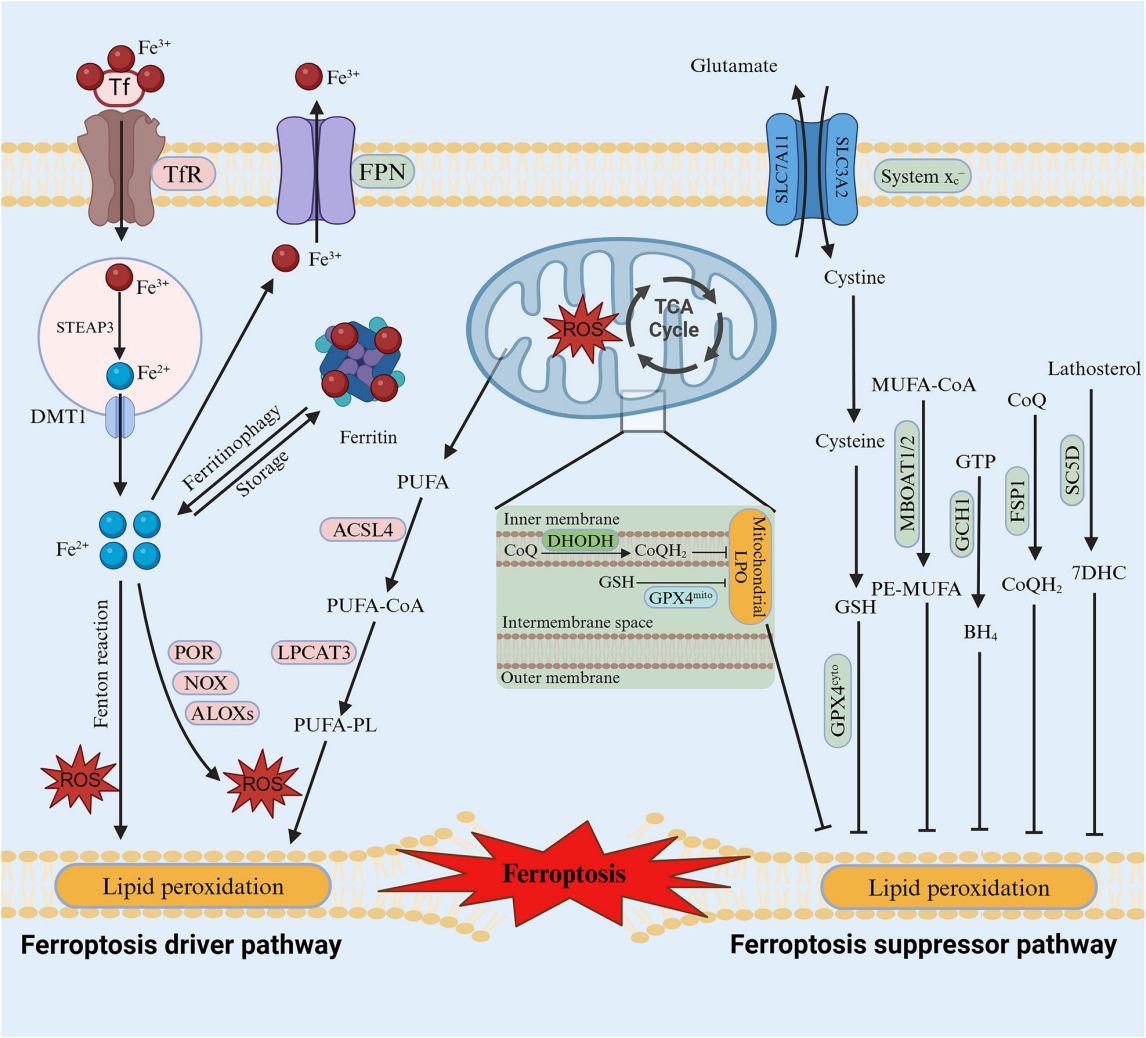
The core mechanisms of ferroptosis. Ferroptosis drive mechanisms: including lipid peroxidation, mitochondrial metabolism and iron overload; Ferroptosis defense mechanisms: including the GPX4-GSH axis, the FSP1-CoQ_10_-H_2_ system, the GCH1-BH_4_ system, the DHODH-CoQH_2_ system, the MBOAT1/2-MUFA system and the SC5D-7-DHC axis. Created with BioRender.com.

Ferroptosis is an iron-dependent lipid peroxidation (LPO)-driven type of cell death, it has a complex regulatory mechanism. The driving system and defense system of ferroptosis are in dynamic equilibrium through a series of complex regulations, and an imbalance will lead to the accumulation of lethal lipid peroxides in the cell membrane, with subsequent rupture of the cell membrane and the occurrence of ferroptosis in the cell [26, 27]. The driving system of ferroptosis mainly includes iron overload, mitochondrial dysfunction, and abnormal lipid metabolism, while the defense system of ferroptosis is more complex, mainly including SLC7A11-GPX4 axis, the FSP1-CoQH_2_ system, the GCH1-BH_4_ system, the DHODH-CoQH_2_ system, the SC5D-7-DHC axis and the MBOAT1/2-MUFA system. Many excellent reviews have detailed the regulatory mechanisms of ferroptosis [16, 20, 28, 29], so here we briefly describe the core mechanisms of ferroptosis and plotted in Fig. 1.

### Ferroptosis drive mechanisms

#### Abnormal lipid metabolism

LPO is the process by which lipids such as polyunsaturated fatty acids (PUFAs) are peroxidized in cell membranes [30, 31]. LPO generates cytotoxic substances such as lipid free radicals and malondialdehyde, which lead to alterations in the fluidity and permeability of cell membranes, and ultimately to cell rupture and death by destroying the phospholipid bilayer of the cell membrane [32]. Peroxidation of PUFAs is a direct and major cause of the occurrence of ferroptosis [33, 34]. PUFAs are converted to PUFA-phospholipids (PUFA-PLs) in the presence of lysophosphatidylcholine acyltransferase 3 (LPCAT3) and acyl-coenzyme A synthase long-chain family member 4 (ACSL4) [35]. PUFA-PLs can be directly driven by iron-dependent Fenton reactions to non-enzymatic LPO; in addition, PUFA-PLs can be facilitated by enzymatic reactions mediated by the oxidoreductases cytochrome P450 reductase (POR), lipoxygenase (LOX), and NADPH oxidase (NOX), and ultimately, cytosolic LPOs induce the onset of ferroptosis [36, 37].

#### Iron overload

Iron in the body is in dynamic equilibrium through a series of complex regulations, if the homeostatic imbalance of iron leads to intracellular iron overload, it will induce ferroptosis. There are two main sources of intracellular Fe^2+^. Firstly, extracellular Fe^3+^ can enter the cell via transferrin and the transferrin receptor, then the Fe^3+^ is converted to Fe^2+^ via STEAP in DMT1. In addition, intracellular Fe^2+^ can also come from ferritin autophagy. Similarly, excess Fe^2+^ can induce cellular ferroptosis through two pathways. First, Fe^2+^ can induce ferroptosis by mediating the non-enzymatic Fenton reaction that directly promotes the peroxidation of PUFA-PL [38, 39]. In addition, Fe^2+^ serves as an indispensable cofactor for iron-dependent lipid peroxidase enzymes (such as LOX and POR) to initiate the peroxidation of PUFA-PL in the cell membrane [40, 41].

#### Mitochondrial dysfunction

Mitochondria can influence ferroptosis by regulating multiple metabolic pathways [42]. First, reactive oxygen species (ROS) in cells are predominantly derived from mitochondria, and ROS are a prerequisite for LPO and ferroptosis [43]. Fe^2+^ in mitochondria can undergo a Fenton reaction with H_2_O_2_ to generate ROS, which promotes PUFA-PL peroxidation to induce ferroptosis [44, 45]. In addition, mitochondria are a major source of ATP [46, 47]. Under conditions of ATP depletion, PUFA-PL synthesis will be inhibited, which inhibits ferroptosis; conversely, acetyl coenzyme A carboxylase is activated under conditions of ATP sufficiency, which promotes PUFA-PL synthesis and ferroptosis [48, 49]. Finally, excess free Fe^2+^ will induce mitochondrial autophagy, leading to mitochondrial rupture and the entry of large amounts of ROS, Fe^2+^, and lipid peroxides into the cytoplasm, thereby exacerbating ferroptosis [50]. In conclusion, mitochondria can regulate ferroptosis by modulating lipid metabolism, iron metabolism, and ROS production.

### Ferroptosis defense mechanisms

#### SLC7A11-GPX4 axis

The SLC7A11-GPX4 axis was the first defense mechanism against ferroptosis to be identified [35]. GPX4 has three isoforms: cytoplasmic, cytosolic, and mitochondrial, among them, cytoplasmic and mitochondrial GPX4 plays a key role in the regulation of ferroptosis [17]. GPX4 converts toxic LPO products to non-toxic PUFA-PL alcohols while oxidizing reduced glutathione to oxidized glutathione, thereby inhibiting lipid peroxide accumulation and ferroptosis [51, 52]. In addition, this axis is closely associated with Xc^-^, an amino acid reverse transporter consisting of solute carrier family 7 member 11 (SLC7A11) and solute carrier family 3 member 2 (SLC3A2) protein, which reverse transfers glutamate and cystine in a 1:1 ratio, while reducing cystine to cysteine and facilitating the translocation of cysteine, the raw material for glutathione synthesis, into the cell [53, 54]. The SLC7A11-GPX4 axis is the main suppression axis for ferroptosis, and blocking it induces ferroptosis [53–55].

#### Others

The ferroptosis suppressor protein 1 (FSP1)-ubiquinol (CoQH_2_) system is another mechanism for inhibiting LPO and ferroptosis. FSP1 converts ubiquinone (CoQ) to CoQH_2_, and CoQH_2_ prevents ferroptosis by suppressing LPO [56, 57]. In addition, with the intensification of ferroptosis studies, more and more inhibition mechanisms of ferroptosis have been identified. For example, the GCH1-BH_4_ system [58], the DHODH-CoQH_2_ system [59], the SC5D-7-DHC axis [60, 61], and the MBOAT1/2-MUF system [62], the roles of these ferroptosis inhibitory mechanisms in ferroptosis have been gradually revealed.

## NcRNA and its regulatory role in ferroptosis

Many studies have suggested that ncRNAs are key regulatory mediators of ferroptosis, ncRNAs can affect ferroptosis by regulating LPO, mitochondrial metabolism, iron metabolism, and the SLC7A11-GPX4 axis, thus affecting the biological behaviors of a wide range of tumors [63, 64]. Current studies on the regulation of ferroptosis have focused on lncRNAs, miRNAs, and circRNAs.

MiRNAs were first reported in 1993 in the Caenorhabditis elegans and are one of the most studied ncRNAs [65], with a length of about 20-25 nucleotides, which play important regulatory roles by modulating target genes and post-transcriptional processes [66, 67]. They modulate target genes mainly by binding to the 3’UTR of mRNAs [68, 69]. The binding of miRNAs to mRNAs of target genes can result in reduced stability of mRNAs or translational repression [70, 71]. In tumors, miRNAs play important roles as cancer genes or cancer suppressor genes in regulating tumor cell growth, proliferation, metastasis, and treatment resistance [72–74]. Several studies have shown that miRNAs can regulate tumor cell ferroptosis by targeting ferroptosis-related genes or proteins. For example, miR-128-3p can induce prostate cancer ferroptosis by inhibiting SLC7A11 [75], miR-424-5p can inhibit ACSL4 to suppress ovarian cancer ferroptosis [76], and miR-324-3p can enhance breast cancer ferroptosis by inhibiting GPX4 [77].

LncRNAs are one of the most common ncRNAs that are not directly involved in protein coding [78]. However, lncRNAs can interact with DNA, RNAs, and proteins to form complex complexes, through which lncRNAs can indirectly be involved in the regulation of genetic information, including the regulation of transcription, mRNA stability, and translation process [79, 80]. Numerous studies have shown its key role in the development of various tumors [81–83]. Recent evidence suggests that lncRNAs play a key regulatory role in tumor cell ferroptosis. For example, lncRNA PMAN inhibits ferroptosis in gastric cancer by up-regulating SLC7A11, whereas lncRNA HEPFAL promotes ferroptosis in hepatocellular carcinoma by down-regulating SLC7A11 [84, 85].

CircRNAs are covalently closed and highly conserved ncRNAs formed by reverse splicing of the 3’ and 5’ ends of mRNAs [86]. Unlike common linear RNAs, the closed-loop structure of circRNAs makes them more stable and less susceptible to degradation [87–90]. CircRNAs can act as sponges of miRNAs to bind competitively with miRNAs, which in turn affects the transcription and stability of miRNAs and thus exerts biological roles [91–93]. In addition, circRNAs can also directly bind to proteins to regulate ferroptosis. CircRNAs have been shown to play important regulatory roles in tumors, and much evidence suggests that circRNAs can influence tumor advancement by regulating ferroptosis. For example, circIL4R can enhance GPX4 by targeting miR-541-3p, thereby inhibiting ferroptosis in hepatocellular carcinoma [94], circLRFN5 promotes glioblastoma ferroptosis by down-regulating PRRX2 [95].

## Current status of research on ferroptosis in CRC

Ferroptosis has become a hot topic in the field of oncology. The role of ferroptosis in CRC is being extensively studied, existing studies have shown that aberrant regulation of ferroptosis is often accompanied in CRC cells [96, 97], and that the aberrant regulation of ferroptosis influences a series of malignant behaviors, such as survival, proliferation, metastasis, and drug resistance of CRC cells [98–100]. In addition, ferroptosis-related biomarkers are effective tools for the diagnosis and prognostic assessment of CRC [101], and ferroptosis has also emerged as a potential therapeutic target for CRC [102].

### The role of ferroptosis in CRC progression

The role of ferroptosis in CRC has been gradually elucidated in recent years. First, ferroptosis can suppress the survival and proliferation of CRC cells. When ferroptosis occurs in tumor cells, LPO leads to cell membrane damage, intracellular substance leakage, and cell metabolism disorders, thus affecting the survival and proliferation ability of cells [99]. In addition, ferroptosis can activate some signaling pathways in cells, such as the p53 signaling pathway, which can further inhibit cell proliferation [103, 104]. Second, ferroptosis affects the invasive and metastatic abilities of CRC cells. For example, adenomatous polyposis coli membrane recruitment 1 deficiency facilitates distant metastasis of CRC by suppressing SLC7A11 and ferritin light chain-mediated ferroptosis [105]; krüppel-like factor 2 induces ferroptosis in CRC through the PI3K/AKT signaling pathway, thereby inhibiting its metastatic ability [106]. Finally, ferroptosis has also been closely associated with CRC drug resistance [107, 108]. Ubiquitin protein ligase E3 component n-recognin 5 inhibits ferroptosis through the smad3/SLC7A11 pathway, thereby leading to chemotherapy resistance of CRC cells [109]. Similarly, cytochrome P450 1B1 induced anti-PD-1 resistance in CRC by inhibiting ferroptosis [110].

### Role of ferroptosis in the diagnosis and prognosis of CRC

CRC is a highly malignant tumor. Currently, there are no accurate tools to diagnose CRC or assess its prognosis. Blood tests, fecal tests, colonoscopy, and computed tomography are currently the main diagnostic methods for CRC, but these tests do not apply to all patients due to sensitivity, specificity, economic cost, and patient tolerance [111, 112]. Therefore, the development of simple and efficient diagnostic tools is urgent. Some studies have shown that ferroptosis-related biomarkers are useful for the early diagnosis of CRC [113, 114]. Transferrin test strips are a highly sensitive diagnostic tool for detecting not only cancer but also pre-cancerous lesions, with a high degree of accuracy [114]. In addition, there is currently a lack of effective prognostic markers for CRC patients, and studies have shown that ferroptosis-related ncRNAs can effectively predict the prognosis of CRC patients [115, 116]. For example, the prognostic model based on 10 ferroptosis-related ncRNAs can accurately assess the prognosis and survival time of CRC patients [117].

### Role of ferroptosis in the treatment of CRC

Studies have shown that anti-tumor drugs can act by inducing ferroptosis in tumor cells. For example, cisplatin can trigger ferroptosis through induced depletion of reduced glutathione and inactivation of glutathione peroxidase [21, 118]. In addition, drugs such as 5-FU and sulfasalazine have been found to induce ferroptosis [119, 120]. Therefore, ferroptosis combined with radiotherapy, chemotherapy, and immunotherapy can improve CRC outcomes [121]. Radiotherapy can directly damage the cellular DNA and cellular membranes and also induce oxidative stress, which synergistically with ferroptosis enhances the killing effect on cancer cells [122]. Chemotherapeutic agents can promote ferroptosis by increasing the level of intracellular oxidative stress [119]. Immunotherapy kills cancer cells by activating the body’s immune system, while ferroptosis can alter the tumor microenvironment and enhance the recognition and killing ability of immune cells [123–125]. In addition, targeting ferroptosis reversed the drug resistance of CRC cells [126, 127]. It was shown that inhibition of the SLC7A11-GPX4 axis induced ferroptosis reversed CRC resistance to 5-FU [128]. Similarly, 3-bromopyruvate overcame cetuximab resistance in CRC cells by inducing ferroptosis [129]. These studies suggest that ferroptosis is of great research value in the treatment of CRC.

## NcRNAs affect CRC progression by regulating ferroptosis

**Fig. 2:**
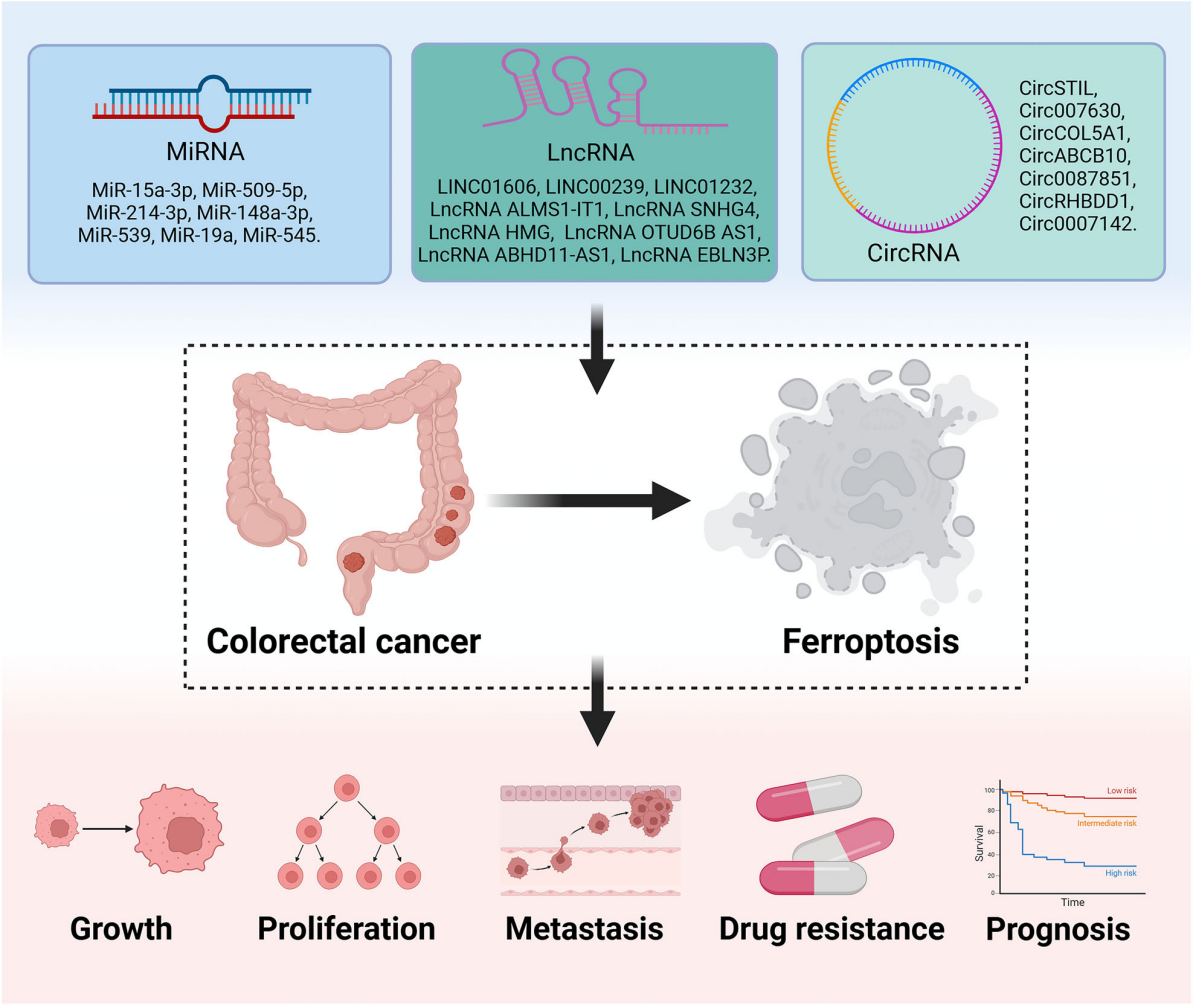
Roles of ncRNA-regulated ferroptosis in colorectal cancer. NcRNA affects colorectal cancer cell growth, proliferation, metastasis, and drug resistance by regulating ferroptosis and can be used to predict the prognosis of colorectal cancer patients. Created with BioRender.com.

**Fig. 3:**
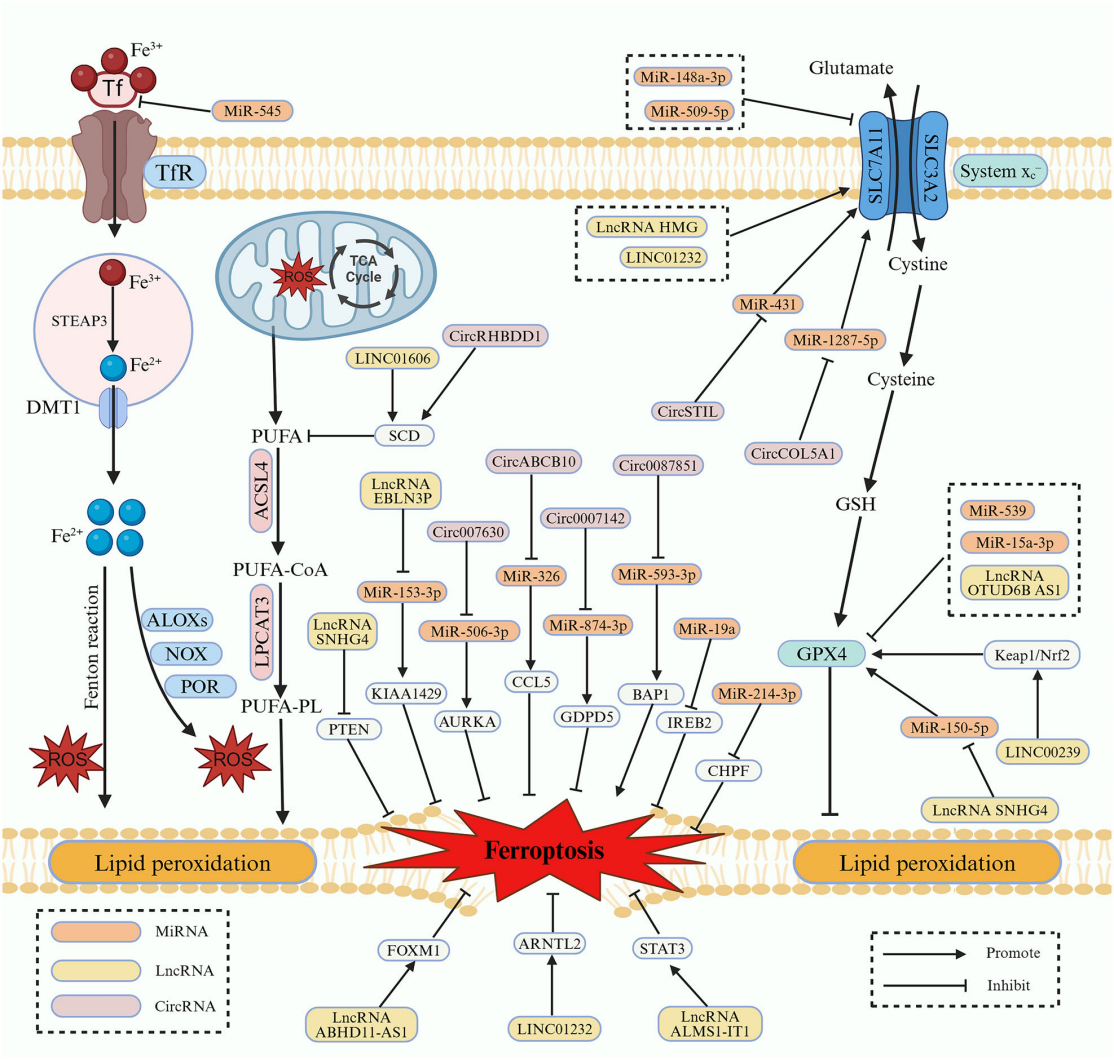
Mechanisms of ncRNA in colorectal cancer ferroptosis. NcRNAs can affect ferroptosis in colorectal cancer cells by regulating iron metabolism, mitochondrial metabolism and the SLC7A11-GPX4 axis or directly. Created with BioRender.com.

Many studies have demonstrated the regulatory role of ncRNAs in the ferroptosis of tumor cells, and ncRNAs can affect malignant behaviors such as tumorigenesis, progression, and drug resistance by regulating ferroptosis [64, 130–132]. Here, we summarize the roles of ncRNAs including miRNAs, lncRNAs, and circRNAs in CRC ferroptosis (Fig. 2), and summarize the mechanisms of ncRNAs in CRC ferroptosis in Fig. 3.

### Role of miRNAs in CRC ferroptosis

Recent evidence suggests that miRNAs can regulate ferroptosis in tumors, especially CRC, and thus affect tumor progression [28, 133–135]. For example, a recent study has suggested that miR-148a-3p can target SLC7A11 through the ACSL4/TFRC/Ferritin axis to promote LPO and ferroptosis, but low miR-148a-3p expression inhibited ferroptosis and promoted survival of CRC cells [135]. Similarly, miR-509-5p inhibited SLC7A11 expression, thereby promoting ferroptosis and inhibiting survival of CRC cells, but low miR-509-5p expression in CRC cells and tissues led to the opposite result [136]. MiR-214-3p was similarly reduced in expression in CRC and correlated with adverse outcomes in CRC patients, it was shown that miR-214-3p directly targets chondroitin polymerizing factor and negatively regulates its expression in CRC cells, which in turn promotes ferroptosis and inhibits the growth of CRC cells [137]. On the contrary, miR-545 expression was significantly up-regulated in CRC cells, but up-regulated miR-545 could reduce LPO and iron accumulation by inhibiting transferrin, thereby inhibiting ferroptosis and promoting the survival and growth of CRC cells in vivo and in vitro [138]. Similarly, miR-526b-5p was highly expressed in CRC tissues and correlated with poor patient prognosis. MiR-526b-5p up-regulated the expression of SLC7A11 and GPX4 by inhibiting P53, which suppressed 5-FU-induced LPO and ferroptosis, and enhanced the survival, invasion, migration, and chemotherapy resistance of CRC cells [139]. Earlier evidence suggests that miR-539 could inhibit the progression of CRC cells [140, 141], but its mechanism of action whether it is related to ferroptosis is unclear. A study found that miR-539 can regulate the expression of tumor necrosis factor-α-induced protein 8, which in turn activated the SAPK/JNK pathway, decreased the expression of GPX4, and promoted the occurrence of ferroptosis and inhibited the proliferation of CRC cells, in contrast, down-regulation of miR-539 expression in CRC promotes CRC development [142]. MiR-19a is a carcinogenic miRNA that is overexpressed in CRC and is associated with the malignant progression of CRC [143, 144]. A recent study has demonstrated that miR-19a inhibits ferroptosis and promotes the proliferation of CRC cells through suppression of iron-responsive element-binding protein 2 [145]. In addition, a study found that miR-15a-3p can directly inhibit GPX4, resulting in tumor cells with increased Fe^2+^ levels and ROS, which in turn contributed to ferroptosis and suppressed the proliferation, invasion, and migration of tumor cells, however, miR-15a-3p expression was down-regulated in CRC cells, which led to the development of CRC [146]. The regulatory role of miRNAs in CRC ferroptosis is summarized in Table 1.

**Table 1:** The regulatory role of ncRNA in colorectal cancer ferroptosis.

NcRNA	Expression	Mechanism	Ferroptosis	Outcome	Ref
MiR-148a-3p	Down-regulated	Regulates the SLC7A11/ACSL4/TFRC/Ferritin axis	↑	Inhibits survival	[135]
MiR-509-5p	Down-regulated	Inhibits SLC7A11 and increases iron levels	↑	Inhibits survival	[136]
MiR-214-3p	Down-regulated	Regulates the miR-214-3p/CHPF axis	↑	Inhibits growth	[137]
MiR-545	Up-regulated	Inhibits Transferrin	↓	Promotes survival and growth	[138]
MiR-526b-5p	Up-regulated	Up-regulation of SLC7A11 and GPX4 expression by inhibition of P53	↓	Promotes survival, migration and drug resistance	[139]
MiR-539	Down-regulated	Regulates the TIPE/SAPK/JNK axis	↑	Inhibits growth	[142]
MiR-19a	Up-regulated	Inhibits IREB2	↓	Promotes proliferation	[145]
MiR-15a-3p	Down-regulated	Inhibits GPX4	↑	Inhibits proliferation, invasion, and migration	[146]
LINC01232	Up-regulated	Enhancement of ARNTL2 transcriptional activity	↓	Promotes proliferation	[149]
LncRNA SNHG4	Up-regulated	Regulates the miR-150-5p/c-Myb/CDO1 axis	↓	Promotes proliferation	[153]
LINC00239	Up-regulated	The Nrf2 signaling pathway is activated by interaction with Keap1	↓	Promotes proliferation	[154]
LncRNA MIR210HG	Up-regulated	By binding to PCBP1 to stabilize its expression, thereby inhibiting iron-dependent LPO	↓	Promotes proliferation	[155]
LncRNA ABHD11-AS1	Up-regulated	Regulates the TRIM21/IGF2BP2/FOXM1 axis	↓	Promotes proliferation and migration	[160]
LncRNA ALMS1-IT1	Up-regulated	Promotes STAT3 phosphorylation	↓	Promotes growth and metastasis	[162]
LINC01606	Up-regulated	It interacts with miR-423-5p to up-regulate SCD1 and activate the wnt/β-catenin pathway	↓	Promotes growth, invasion, and stemness	[164]
LINC02159	Up-regulated	Stabilization of FOXC2 expression by deubiquitination	↑	Inhibits proliferation, migration, and invasion	[165]
LncRNA CRCMSL	Up-regulated	By down-regulating the expression of ACC1, the accumulation of PUFAs is increased, which in turn increases the level of LPO.	↑	Inhibits invasion and migration	[166]
LncRNA OTUD6B-AS1	Down-regulated	Stabilized TRIM16 and downregulated GPX4 by binding to HuR	↑	Inhibits radiotherapy resistance	[167]
LncRNA SNHG4	Up-regulated	Targets PTEN	↓	Promotes drug resistance	[170]
LncRNA HMG	Up-regulated	LncRNA HMG activated by the wnt/β-catenin pathway up-regulates the expression of SLC7A11 and VKORC1L1 by inhibiting p53	↓	Promotes drug resistance	[171]
LncRNA EBLN3P	Up-regulated	KIAA1429 upregulates lncRNAEBLN3P, and lncRNAEBLN3P in turn upregulates KIAA1429 through competitive binding with miR-153-3p	↓	Promotes radiotherapy resistance	[172]
CircABCB10	Up-regulated	Regulates the miR-326/CCL5 axis	↓	Promotes growth	[175]
CircSTIL	Up-regulated	Regulates the miR-431/SLC7A11 axis	↓	Promotes proliferation	[176]
Circ0075829	Up-regulated	Regulates the miR-330-5p/TCF4 axis	↓	Promotes proliferation	[177]
Circ0007142	Up-regulated	Regulates the miR-874-3p/GDPD5 axis	↓	Promotes proliferation and survival	[180]
CircCOL5A1	Up-regulated	Regulates the miR-1287-5p/SLC7A11 axis	↓	Promotes proliferation and invasion	[183]
CircRHBDD1	Up-regulated	Regulates the ELAVL1/SCD axis	↓	Promotes proliferation and migration	[184]
Circ007630	Up-regulated	Regulates the miR-506-3p/AURKA axis	↓	Promotes proliferation, invasion, and migration	[188]
Circ0087851	Down-regulated	Regulates the miR-593-3p/BAP1 axis	↑	Inhibits growth, migration, and invasion	[189]

*CHPF* chondroitin polymerizing factor, *SLC7A11* solute carrier family 7 member 11, *ACSL4* acyl-coenzyme a synthase long-chain family member 4, *TFRC* transferrin receptor, *IREB2* iron-responsive element- binding protein 2, *TIPE* tumor necrosis factor-α-induced protein 8, *TRIM21* tripartite motif containing 21, *IGF2BP2* insulin-like growing factor 2 mRNA-binding protein 2, *FOXM1* forkhead box M1, *CDO1* cysteine dioxygenase 1, *SCD1* stearoyl-CoA desaturase 1, *VKORC1L1* vitamin k oxidoreductase like 1, *AURKA* aurora kinase A, *ACC1* acetyl coenzyme A carboxylase 1, *PCBP1* poly(rC) binding protein 1, *CCL5* C-C motif chemokine ligand 5, GDPD5 glycerophosphodiester phosphodiesterase domain containing 5, *BAP1* BRCA1 associated protein 1.

### Role of lncRNAs in CRC ferroptosis

Recently, the role of some lncRNAs in CRC ferroptosis has been revealed, and thus, targeting lncRNAs may have great therapeutic value in CRC patients. Previous evidence demonstrated that LINC01232 significantly promotes the proliferation, migration, and angiogenesis of CRC and pancreatic cancer [147, 148]. However, whether LINC01232 acts by regulating ferroptosis is unknown. A recent study has shown that LINC01232 interacts with p300 to enhance H3K27ac modifications on the ARNTL2 promoter, which promotes the transcriptional activity of ARNTL2, thereby inhibiting ferroptosis in CRC and promoting the proliferation of CRC cells [149]. The small nucleolar RNA host genes (SNHG) are a type of lncRNA, that is strongly associated with malignant behavior and poor prognosis of tumors [150–152]. A recent study has shown that SNHG4 is overexpressed in CRC cells, it can increase the level of c-Myb protein by inhibiting miR-150, the overexpressed c-Myb can reduce the expression level of CDO1 and up-regulate GPX4, then it can inhibit ferroptosis and promote the proliferation of CRC cells [153]. Similarly, it was demonstrated that LINC00239, an inhibitor of ferroptosis, is overexpressed in CRC and is related to an adverse prognosis of CRC patients, LINC00239 activates the NF-E2-related factor 2 signaling pathway by interacting with Kelch-like ECH-associated protein 1, which inhibits ferroptosis and promotes proliferation of CRC cells [154]. Another study found that lncRNA MIR210HG is overexpressed in CRC tissues and cell lines, it was closely associated with poor patient prognosis. LncRNA MIR210HG stabilizes its expression by binding to poly(rC) binding protein 1 and inhibits iron-dependent LPO, thereby promoting CRC cell proliferation by inhibiting ferroptosis [155]. In addition, ferroptosis regulated by lncRNAs can also affect CRC metastasis. LncRNA ABHD11-AS1 is overexpressed in multiple cancers and associated with patient progression [156–159], but the mechanisms are unclear. A recent study has shown that overexpression of ABHD11-AS1 in CRC was negatively related to patient survival, further studies showed that ABHD11-AS1 can enhance the stability of FOXM1 by binding to the m⁶A-reading protein IGF2BP2, thereby forming an ABHD11-AS1/FOXM1 positive feedback loop. In addition, ABHD11-AS1 promotes the interaction of TRIM21 with IGF2BP2, mediating the ubiquitination degradation of IGF2BP2 and indirectly regulating FOXM1 stability. Ultimately, it inhibits ferroptosis and promotes CRC proliferation and migration [160]. Previous evidence suggests that expression of the lncRNA ALMS1-IT1 may be associated with ferroptosis [161]. A recent study demonstrated that ALMS1-IT1 was significantly up-regulated in CRC tissues and inhibited ferroptosis by promoting stat3 phosphorylation, which promoted CRC cell growth and metastasis [162]. LINC01606 has been shown to promote ferroptosis by triggering the wnt/β-catenin pathway to promote malignant behavior in gastric cancer, but its regulatory role in CRC is unknown [163]. A recent study has shown that LINC01606 is overexpressed in CRC cells and tissues, it can up-regulate stearoyl-CoA desaturase1 (SCD1) through interaction with miR-423-5p, then activate the wnt/β-catenin pathway and inhibits the ferroptosis of CRC cells, Ultimately, it promoted CRC growth, invasion, and stemness [164].

LncRNA can also promote CRC ferroptosis. For example, LINC02159 can stabilize the expression of FOXC2 by deubiquitination, thereby inhibiting CRC proliferation, migration and invasion by promoting CRC ferroptosis, but the mechanism is not fully understood [165]. Acetyl Coenzyme A carboxylase 1 (ACC1) is a key enzyme in fatty acid synthesis, and lncRNA CRCMSL can inactivate ACC1 by regulating its phosphorylation, thereby reducing the production of MUFAs and increasing the proportion of PUFAs. Accumulation of PUFAs enhances the level of LPO and activates ferroptosis, which inhibits the invasion and migration ability of CRC cells [166]. Similarly, another study found that the lncRNA OTUD6B-AS1 was decreased in CRC cells and tissues, and more importantly, its low expression was more pronounced in radioresistant CRC cells. Further studies found that OTUD6B-AS1 can bind HuR to stabilize TRIM16, enhancing GPX4-mediated ferroptosis and improving CRC radiotherapy sensitivity, whereas low expression of OTUD6B-AS1 resulted in CRC radiotherapy resistance [167].

In addition, many studies have reported that lncRNAs are related to tumor therapy resistance [168, 169], but whether ferroptosis-associated lncRNAs are associated with CRC therapy resistance has not been revealed. SNHG4 not only affects CRC cell proliferation by regulating ferroptosis but also has been associated with chemotherapy resistance in CRC cells. It was found that SNHG4 expression was significantly increased in oxaliplatin-resistant CRC cells compared to non-resistant cells, and mechanistic studies suggest that overexpressed SNHG4 decreases mRNA stability in tumor cells by targeting PTEN, thereby inhibiting ferroptosis and promoting oxaliplatin resistance [170]. Similarly, it was found that the lncRNA HMG is elevated in CRC and correlated with chemotherapy resistance in CRC patients, lncRNA HMG is activated by the wnt/β-catenin signaling pathway, which up-regulates ferroptosis inhibitors SLC7A11 and VKORC1L1 through inhibits p53, in turn suppresses ferroptosis and chemotherapy resistance in CRC cells [171]. In addition to chemotherapy resistance, lncRNAs are also closely associated with CRC radiotherapy resistance. It has been shown that m6A methyltransferase KIAA1429-mediated m6A modification in CRC cells up-regulates lncRNA EBLN3P, and the up-regulated lncRNA EBLN3P in turn enhances the expression of KIAA1429 through competitive binds to miR-153-3p, which ultimately inhibits ferroptosis in CRC cells and enhanced its radiotherapy resistance [172]. Overall, these pieces of evidence suggest that lncRNAs can affect CRC progression and drug resistance by regulating ferroptosis, and targeting ferroptosis-related lncRNAs has great research value in the treatment of CRC. The regulatory role of lncRNAs in CRC ferroptosis is summarized in Table 1.

### Role of circRNA in CRC ferroptosis

Many studies have suggested that circRNAs can influence the progression of various malignancies by regulating ferroptosis in tumor cells [64, 173, 174]. Several recent studies have also reported that circRNA affects CRC progression by regulating ferroptosis. For example, a study found that circABCB10, whose expression is up-regulated in CRC, up-regulates C-C motif chemokine ligand 5 via miR-326, which reduces intracellular ROS and Fe^2+^ accumulation, inhibits CRC cell ferroptosis and promotes their growth [175]. Similarly, it was demonstrated that circSTIL expression levels are remarkably increased in CRC cells and tissues and are strongly related to adverse prognosis in CRC patients, circSTIL inhibits ferroptosis and promotes the proliferation of CRC cells via the miR-431/SLC7A11 axis [176]. In addition, circ0075829, which is highly expressed in colon cancer, can up-regulate TCF4 expression by sponging miR-330-5p, thereby inhibiting ferroptosis and promoting proliferation in colon cancer [177]. Circ0007142 has been suggested to be an oncogenic molecule in CRC, but whether it can affect CRC by regulating ferroptosis remains unknown [178, 179]. A recent study has shown that circ0007142 sponges and adsorbs miR-874-3p, relieving the inhibition of miR-874-3p on the target gene glycerophosphodiester phosphodiesterase domain containing 5. Thus, it can inhibit ferroptosis and promote the survival of CRC cells [180]. Similarly, some studies have suggested that the aberrant expression of circCOL5A1 is closely related to human diseases [181, 182], however, the role of circCOL5A1 in CRC is unclear. Wang et al. found that overexpression of circCOL5A1 in CRC was related to the adverse prognosis of CRC patients, and further mechanistic studies showed that circCOL5A1 inhibited ferroptosis of CRC cells through miR-1287-5p/SLC7A11 axis, thus promoted CRC cell proliferation and invasion [183]. Furthermore, circRNA regulation of ferroptosis also affects CRC metastasis. Recent evidence has suggested that circRHBDD1 expression is significantly up-regulated in CRC cells and tissues, circRHBDD1 up-regulates the ferroptosis inhibitor SCD in an ELAVL1-dependent manner, which in turn suppresses ferroptosis and facilitates migration and proliferation of CRC cells [184]. Aurora kinase A (AURKA), a key gene for ferroptosis, is overexpressed in a wide range of tumors and correlates with the malignant behavior of tumors [185–187]. A recent study has found that AURKA is the target of miR-506-3p, and circ007630 can inhibit ferroptosis through the miR-506-3p/AURKA pathway in CRC and facilitates the proliferation, invasion, and migration of CRC cells [188]. Similarly, it was found that low expression of circ0087851 in CRC was closely correlated with poor prognosis in patients, circ0087851 could promote ferroptosis through miR-593-3p/BAP1 axis and inhibit the growth, migration, and invasion of tumor cells, but the low expression of circ0087851 led to the malignant progression of CRC [189]. The regulatory role of circRNAs in CRC ferroptosis is summarized in Table 1.

## Ferroptosis-associated ncRNA is a biomarker for CRC

With the rapid development of molecular biology and transcriptomics technologies, the research on ncRNAs has become a current research hotspot. NcRNAs have been shown to play essential roles in cancer occurrence, development, treatment, and prognosis [13, 190, 191]. Ferroptosis-associated ncRNAs are not only associated with tumor cell growth, proliferation, and metastasis but are also used to predict patient prognosis as well as the selection of therapeutic regimens [115, 161, 192].

As potential biomarkers, ncRNAs are important in the diagnosis, treatment, and prognosis of a wide range of tumors, including CRC [193, 194]. Recently, many studies have demonstrated that ferroptosis-associated ncRNAs, especially lncRNAs, can be used as potential biomarkers for CRC to predict patient prognosis and sensitivity to drugs [195]. For example, Wu et al. constructed four models of ferroptosis-associated lncRNAs that can be used to predict the treatment response and clinical outcomes in CRC patients, which has a stronger ability to predict patient prognosis than traditional TNM staging and is easier to test in patients [196]. Similarly, Chen et al. successfully screened 6 CRC ferroptosis-related lncRNAs, which can be used as new biomarkers for CRC to predict patient prognosis [116]. In addition, Cai et al. successfully screened 7 ferroptosis-related lncRNAs for the assessment of survival in CRC patients, and Cox multifactorial analysis showed that these lncRNAs were independent prognostic factors [197]. Xu et al. constructed a Prognostic Signature model consisting of 8 ferroptosis-related lncRNAs, which not only independently predicted CRC patients’ prognosis, but also predicted their risk of recurrence and metastasis [198]. In addition to predicting the prognosis of CRC patients, ferroptosis-associated lncRNAs can also be useful in assessing patient response to therapy. For example, a study showed that a model consisting of five ferroptosis-associated lncRNAs could be used to accurately predict immunotherapy response in CRC patients [199]. Similarly, li et al. screened a total of 25 ferroptosis-associated lncRNAs for use in a risk assessment model, which not only significantly outperformed common clinicopathologic features in predicting overall survival in CRC patients, but also could be used to predict patients’ response to immunotherapy [101]. In addition to immunotherapy, the prediction model constructed by Chen et al. based on 15 ferroptosis-associated lncRNAs could also be used to assess patients’ sensitivity to chemotherapy [200]. In addition to lncRNAs, miRNAs also have good predictive ability for CRC patients. A survival prediction model composed of 14 ferroptosis-associated miRNAs showed high accuracy in predicting the survival of CRC patients [201].

These studies suggest that ferroptosis-associated ncRNAs could serve as potential markers of CRC progression, prognosis, individualized therapy, and drug resistance. However, most of the current studies have small samples and the specific mechanism of action of these ferroptosis-associated ncRNAs in CRC is still unclear; therefore, more large-sample studies and exploration of the mechanism of these ncRNAs are necessary.

## Conclusions and perspectives

Many studies have demonstrated that ncRNAs can affect CRC progression by regulating ferroptosis. This review first introduces the key mechanisms of ferroptosis and the role of ncRNAs in ferroptosis, and then we summarize the role of ferroptosis in CRC, we then focus on summarizing the effect of ncRNAs regulating ferroptosis on CRC and the recent progress of ferroptosis-related ncRNAs as prognostic biomarkers in CRC patients. Available evidence suggests that ferroptosis regulated by ncRNAs plays a key role in CRC progression, treatment, and drug resistance. Ferroptosis-associated ncRNAs have high accuracy and feasibility as markers to assess the prognosis of CRC patients. More importantly, the ncRNA-ferroptosis axis is a potential target for CRC and has shown great therapeutic potential.

However, studies on the regulatory role of ncRNAs in CRC ferroptosis are in their infancy, and there are many significant challenges to achieving clinical translation. First, the mechanism of ncRNA regulation of ferroptosis in CRC is not well understood. The relevant studies are still relatively few and limited to the most central mechanism of ncRNAs regulating ferroptosis, such as the SLC7A11-GPX4 axis, and it is still unclear whether ncRNAs regulate ferroptosis through other mechanisms. We believe that studies on the role and mechanism of ncRNAs in CRC ferroptosis will be one of the focuses of future research. Second, the role of ncRNA-regulated ferroptosis in CRC is not yet fully understood. Current research is limited to CRC development and drug resistance, and the roles and mechanisms of ncRNA-regulated ferroptosis in the occurrence and treatment of CRC are still unclear. These intricate roles and mechanisms have seriously hindered the clinical application of ferroptosis-associated ncRNAs, and the efficacy and safety of these ncRNAs as therapeutic targets in clinical application are still a concern. Third, previous studies have shown that there are interactions between ferroptosis and other forms of cell death, particularly autophagy, and that ncRNAs may play an important role in these interactions [202, 203]. For example, lncRNA H19 regulates breast cancer ferroptosis by mediating autophagy [204], and circcIARS regulates hepatocellular carcinoma ferroptosis by modulating ALKBH5-mediated autophagy [205], these studies suggest that targeting ncRNAs and autophagy at the same time may be a new direction for treating tumors. Finally, although some studies have suggested that ferroptosis-associated ncRNAs can be exploited to assess the prognosis of CRC patients, most of them have small sample sizes and the sensitivity and specificity have to be confirmed. More large-sample and high-quality studies are needed to validate this idea before clinical application. In addition, there are still some difficulties in the current ncRNA detection methods and purification techniques, as well as the high cost of detection, which will be a major challenge for them to be translated into clinical application [206]. Developing ncRNA detection methods based on the exosomes or nanomaterials can improve the stability of samples, moreover, the application of next-generation sequencing and digital PCR technologies may be the key to solving these problems [207–210]. In conclusion, despite the growing evidence of the great potential of ferroptosis-associated ncRNAs as therapeutic targets and prognostic markers for CRC patients, studies of ferroptosis-associated ncRNAs and CRC are still in the experimental stage, and they still face many problems and challenges in the process of achieving clinical translations.

In summary, recent pieces of evidence suggest that ncRNAs play an essential role in the progression of CRC by regulating ferroptosis. Our review summarizes the recent progress of ncRNA-mediated ferroptosis in CRC, which will deepen our understanding of the ncRNA-ferroptosis-CRC axis and provide direction and reference for future studies.

## References

[1] Sung H, Ferlay J, Siegel RL, Laversanne M, Soerjomataram I, Jemal A, et al. Global Cancer Statistics 2020: Globocan Estimates of Incidence and Mortality Worldwide for 36 Cancers in 185 Countries. CA Cancer J Clin. 2021;71:209–49. 33538338 10.3322/caac.21660

[2] Siegel RL, Giaquinto AN, Jemal A. Cancer Statistics, 2024. CA Cancer J Clin. 2024;74:12–49. 38230766 10.3322/caac.21820

[3] Sadanandam A, Lyssiotis CA, Homicsko K, Collisson EA, Gibb WJ, Wullschleger S, et al. A colorectal cancer classification system that associates cellular phenotype and responses to therapy. Nat Med. 2013;19:619–25. 23584089 10.1038/nm.3175 PMC3774607

[4] Zhang Y, Wang Y, Zhang B, Li P, Zhao Y. Methods and biomarkers for early detection, prediction, and diagnosis of colorectal cancer. Biomed Pharmacother. 2023;163:114786. 37119736 10.1016/j.biopha.2023.114786

[5] Nishihara R, Wu K, Lochhead P, Morikawa T, Liao X, Qian ZR, et al. Long-term colorectal-cancer incidence and mortality after lower endoscopy. N. Engl J Med. 2013;369:1095–105. 24047059 10.1056/NEJMoa1301969 PMC3840160

[6] Zhang J, Wu L, Wang C, Xie X, Han Y. Research progress of long non-coding RNA in tumor drug resistance: a new paradigm. Drug Des Devel Ther. 2024;18:1385–98. 38689609 10.2147/DDDT.S448707 PMC11060174

[7] Hashemi M, Daneii P, Zandieh MA, Raesi R, Zahmatkesh N, Bayat M, et al. Non-coding RNA-mediated n6-methyladenosine (m(6)a) deposition: a pivotal regulator of cancer, impacting key signaling pathways in carcinogenesis and therapy response. Noncoding RNA Res. 2024;9:84–104. 38075202 10.1016/j.ncrna.2023.11.005 PMC10700483

[8] Zhao J, Ma Y, Zheng X, Sun Z, Lin H, Du C, et al. Bladder cancer: non-coding RNAs and exosomal non-coding RNAs. Funct Integr Genomics. 2024;24:147. 39217254 10.1007/s10142-024-01433-9

[9] Liu QL, Zhang Z, Wei X, Zhou ZG. Noncoding RNAs in tumor metastasis: molecular and clinical perspectives. Cell Mol Life Sci. 2021;78:6823–50. 34499209 10.1007/s00018-021-03929-0 PMC11073083

[10] Winkle M, El-Daly SM, Fabbri M, Calin GA. Noncoding RNA therapeutics—challenges and potential solutions. Nat Rev Drug Discov. 2021;20:629–51. 34145432 10.1038/s41573-021-00219-z PMC8212082

[11] Liu J, Chang X, Manji L, Xu Z, Xiao W. Roles of small peptides encoded by non-coding RNAs in tumor invasion and migration. Front Pharm. 2024;15:1442196. 10.3389/fphar.2024.1442196 PMC11439703 39351098

[12] Elimam H, Moussa R, Radwan AF, Hatawsh A, Elfar N, Alhamshry NAA, et al. LncRNAs orchestration of gastric cancer - particular emphasis on the etiology, diagnosis, and treatment resistance. Funct Integr Genomics. 2024;24:175. 39325107 10.1007/s10142-024-01450-8

[13] Li J, Wang X, Wang H. RNA modifications in long non-coding RNAs and their implications in cancer biology. Bioorg Med Chem. 2024;113:117922. 39299080 10.1016/j.bmc.2024.117922

[14] Chen J, Wei Z, Fu K, Duan Y, Zhang M, Li K, et al. Non-apoptotic cell death in ovarian cancer: treatment, resistance and prognosis. Biomed Pharmacother. 2022;150:112929. 35429741 10.1016/j.biopha.2022.112929

[15] Dixon SJ, Lemberg KM, Lamprecht MR, Skouta R, Zaitsev EM, Gleason CE, et al. Ferroptosis: an iron-dependent form of nonapoptotic cell death. Cell. 2012;149:1060–72. 22632970 10.1016/j.cell.2012.03.042 PMC3367386

[16] Lei G, Zhuang L, Gan B. Targeting ferroptosis as a vulnerability in cancer. Nat Rev Cancer. 2022;22:381–96. 35338310 10.1038/s41568-022-00459-0 PMC10243716

[17] Dixon SJ, Olzmann JA. The cell biology of ferroptosis. Nat Rev Mol Cell Biol. 2024;25:424–42. 38366038 10.1038/s41580-024-00703-5 PMC12187608

[18] Zhou QY, Ren C, Li JY, Wang L, Duan Y, Yao RQ, et al. The crosstalk between mitochondrial quality control and metal-dependent cell death. Cell Death Dis. 2024;15:299. 38678018 10.1038/s41419-024-06691-w PMC11055915

[19] Mao ZH, Gao ZX, Pan SK, Liu DW, Liu ZS, Wu P. Ferroptosis: a potential bridge linking gut microbiota and chronic kidney disease. Cell Death Discov. 2024;10:234. 38750055 10.1038/s41420-024-02000-8 PMC11096411

[20] Ru Q, Li Y, Chen L, Wu Y, Min J, Wang F. Iron homeostasis and ferroptosis in human diseases: mechanisms and therapeutic prospects. Signal Transduct Target Ther. 2024;9:271. 39396974 10.1038/s41392-024-01969-z PMC11486532

[21] Guo J, Xu B, Han Q, Zhou H, Xia Y, Gong C, et al. Ferroptosis: a novel anti-tumor action for cisplatin. Cancer Res Treat. 2018;50:445–60. 28494534 10.4143/crt.2016.572 PMC5912137

[22] Lei G, Zhang Y, Koppula P, Liu X, Zhang J, Lin SH, et al. The role of ferroptosis in ionizing radiation-induced cell death and tumor suppression. Cell Res. 2020;30:146–62. 31949285 10.1038/s41422-019-0263-3 PMC7015061

[23] Wang W, Green M, Choi JE, Gijón M, Kennedy PD, Johnson JK, et al. Cd8(+) T cells regulate tumour ferroptosis during cancer immunotherapy. Nature. 2019;569:270–74. 31043744 10.1038/s41586-019-1170-y PMC6533917

[24] Luo Y, Huang Q, He B, Liu Y, Huang S, Xiao J. Regulation of ferroptosis by non‑coding RNAs in the development and treatment of cancer (Review). Oncol Rep. 2021;45:29–48. 33155665 10.3892/or.2020.7836 PMC7709825

[25] Zuo YB, Zhang YF, Zhang R, Tian JW, Lv XB, Li R, et al. Ferroptosis in cancer progression: role of noncoding RNAs. Int J Biol Sci. 2022;18:1829–43. 35342359 10.7150/ijbs.66917 PMC8935228

[26] Li K, Fan C, Chen J, Xu X, Lu C, Shao H, et al. Role of oxidative stress-induced ferroptosis in cancer therapy. J Cell Mol Med. 2024;28:e18399. 38757920 10.1111/jcmm.18399 PMC11100387

[27] Li Y, Li Z, Ran Q, Wang P. Sterols in ferroptosis: from molecular mechanisms to therapeutic strategies. Trends Mol Med. 2024;31:36–49. 10.1016/j.molmed.2024.08.007 39256109

[28] Sun L, Cao H, Wang Y, Wang H. Regulating ferroptosis by non-coding RNAs in hepatocellular carcinoma. Biol Direct. 2024;19:80. 39267124 10.1186/s13062-024-00530-w PMC11391853

[29] Li S, Zhang G, Hu J, Tian Y, Fu X. Ferroptosis at the nexus of metabolism and metabolic diseases. Theranostics. 2024;14:5826–52. 39346540 10.7150/thno.100080 PMC11426249

[30] Peleman C, Francque S, Berghe TV. Emerging role of ferroptosis in metabolic dysfunction-associated steatotic liver disease: revisiting hepatic lipid peroxidation. EBioMedicine. 2024;102:105088. 38537604 10.1016/j.ebiom.2024.105088 PMC11026979

[31] Fujii J, Yamada KI. Defense systems to avoid ferroptosis caused by lipid peroxidation-mediated membrane damage. Free Radic Res. 2023;57:353–72. 37551716 10.1080/10715762.2023.2244155

[32] Ursini F, Maiorino M. Lipid peroxidation and ferroptosis: the role of Gsh and Gpx4. Free Radic Biol Med. 2020;152:175–85. 32165281 10.1016/j.freeradbiomed.2020.02.027

[33] Doll S, Proneth B, Tyurina YY, Panzilius E, Kobayashi S, Ingold I, et al. Acsl4 dictates ferroptosis sensitivity by shaping cellular lipid composition. Nat Chem Biol. 2017;13:91–98. 27842070 10.1038/nchembio.2239 PMC5610546

[34] Kagan VE, Mao G, Qu F, Angeli JP, Doll S, Croix CS, et al. Oxidized arachidonic and adrenic Pes navigate cells to ferroptosis. Nat Chem Biol. 2017;13:81–90. 27842066 10.1038/nchembio.2238 PMC5506843

[35] Wang H, Fleishman JS, Cheng S, Wang W, Wu F, Wang Y, et al. Epigenetic modification of ferroptosis by non-coding RNAs in cancer drug resistance. Mol Cancer. 2024;23:177. 39192329 10.1186/s12943-024-02088-7 PMC11348582

[36] Yan B, Ai Y, Sun Q, Ma Y, Cao Y, Wang J, et al. Membrane damage during ferroptosis is caused by oxidation of phospholipids catalyzed by the oxidoreductases Por and Cyb5r1. Mol Cell. 2021;81:355–69.e10. 33321093 10.1016/j.molcel.2020.11.024

[37] Chu B, Kon N, Chen D, Li T, Liu T, Jiang L, et al. Alox12 is required for P53-mediated tumour suppression through a distinct ferroptosis pathway. Nat Cell Biol. 2019;21:579–91. 30962574 10.1038/s41556-019-0305-6 PMC6624840

[38] Dixon SJ, Stockwell BR. The role of iron and reactive oxygen species in cell death. Nat Chem Biol. 2014;10:9–17. 24346035 10.1038/nchembio.1416

[39] Doll S, Conrad M. Iron and ferroptosis: a still ill-defined liaison. IUBMB Life. 2017;69:423–34. 28276141 10.1002/iub.1616

[40] Yang WS, Kim KJ, Gaschler MM, Patel M, Shchepinov MS, Stockwell BR. Peroxidation of polyunsaturated fatty acids by lipoxygenases drives ferroptosis. Proc Natl Acad Sci USA. 2016;113:E4966–75. 27506793 10.1073/pnas.1603244113 PMC5003261

[41] Koppula P, Zhuang L, Gan B. Cytochrome P450 reductase (Por) as a ferroptosis fuel. Protein Cell. 2021;12:675–79. 33539003 10.1007/s13238-021-00823-0 PMC8403093

[42] Gan B. Mitochondrial regulation of ferroptosis. J Cell Biol 220 2021;220. 10.1083/jcb.202105043 PMC8329737 34328510

[43] Willems PH, Rossignol R, Dieteren CE, Murphy MP, Koopman WJ. Redox homeostasis and mitochondrial dynamics. Cell Metab. 2015;22:207–18. 26166745 10.1016/j.cmet.2015.06.006

[44] Murphy MP. How mitochondria produce reactive oxygen species. Biochem J. 2009;417:1–13. 19061483 10.1042/BJ20081386 PMC2605959

[45] Zheng J, Conrad M. The metabolic underpinnings of ferroptosis. Cell Metab. 2020;32:920–37. 33217331 10.1016/j.cmet.2020.10.011

[46] Friedman JR, Nunnari J. Mitochondrial form and function. Nature. 2014;505:335–43. 24429632 10.1038/nature12985 PMC4075653

[47] Vasan K, Werner M, Chandel NS. Mitochondrial metabolism as a target for cancer therapy. Cell Metab. 2020;32:341–52. 32668195 10.1016/j.cmet.2020.06.019 PMC7483781

[48] Li C, Dong X, Du W, Shi X, Chen K, Zhang W, et al. Lkb1-Ampk axis negatively regulates ferroptosis by inhibiting fatty acid synthesis. Signal Transduct Target Ther. 2020;5:187. 32883948 10.1038/s41392-020-00297-2 PMC7471309

[49] Lee H, Zandkarimi F, Zhang Y, Meena JK, Kim J, Zhuang L, et al. Energy-stress-mediated Ampk activation inhibits ferroptosis. Nat Cell Biol. 2020;22:225–34. 32029897 10.1038/s41556-020-0461-8 PMC7008777

[50] Yu F, Zhang Q, Liu H, Liu J, Yang S, Luo X, et al. Dynamic O-glcnacylation coordinates ferritinophagy and mitophagy to activate ferroptosis. Cell Discov. 2022;8:40. 35504898 10.1038/s41421-022-00390-6 PMC9065108

[51] Seibt TM, Proneth B, Conrad M. Role of Gpx4 in ferroptosis and its pharmacological implication. Free Radic Biol Med. 2019;133:144–52. 30219704 10.1016/j.freeradbiomed.2018.09.014

[52] Brigelius-Flohé R, Flohé L. Regulatory phenomena in the glutathione peroxidase superfamily. Antioxid Redox Signal. 2020;33:498–516. 31822117 10.1089/ars.2019.7905

[53] Xiang S, Yan W, Ren X, Feng J, Zu X. Role of ferroptosis and ferroptosis-related long non’coding RNA in breast cancer. Cell Mol Biol Lett. 2024;29:40. 38528461 10.1186/s11658-024-00560-2 PMC10964675

[54] Koppula P, Zhang Y, Zhuang L, Gan B. Amino acid transporter Slc7a11/Xct at the crossroads of regulating redox homeostasis and nutrient dependency of cancer. Cancer Commun (Lond). 2018;38:12. 29764521 10.1186/s40880-018-0288-x PMC5993148

[55] Yang WS, SriRamaratnam R, Welsch ME, Shimada K, Skouta R, Viswanathan VS, et al. Regulation of ferroptotic cancer cell death by Gpx4. Cell. 2014;156:317–31. 24439385 10.1016/j.cell.2013.12.010 PMC4076414

[56] Bersuker K, Hendricks JM, Li Z, Magtanong L, Ford B, Tang PH, et al. The Coq oxidoreductase Fsp1 acts parallel to Gpx4 to inhibit ferroptosis. Nature. 2019;575:688–92. 31634900 10.1038/s41586-019-1705-2 PMC6883167

[57] Nakamura T, Hipp C, Santos Dias Mourão A, Borggräfe J, Aldrovandi M, Henkelmann B, et al. Phase separation of Fsp1 promotes ferroptosis. Nature. 2023;619:371–77. 37380771 10.1038/s41586-023-06255-6 PMC10338336

[58] Soula M, Weber RA, Zilka O, Alwaseem H, La K, Yen F, et al. Metabolic determinants of cancer cell sensitivity to canonical ferroptosis inducers. Nat Chem Biol. 2020;16:1351–60. 32778843 10.1038/s41589-020-0613-y PMC8299533

[59] Mao C, Liu X, Zhang Y, Lei G, Yan Y, Lee H, et al. Dhodh-mediated ferroptosis defence is a targetable vulnerability in cancer. Nature. 2021;593:586–90. 33981038 10.1038/s41586-021-03539-7 PMC8895686

[60] Freitas FP, Alborzinia H, Dos Santos AF, Nepachalovich P, Pedrera L, Zilka O, et al. 7-dehydrocholesterol is an endogenous suppressor of ferroptosis. Nature. 2024;626:401–10. 38297129 10.1038/s41586-023-06878-9

[61] Li Y, Ran Q, Duan Q, Jin J, Wang Y, Yu L, et al. 7-dehydrocholesterol dictates ferroptosis sensitivity. Nature. 2024;626:411–18. 38297130 10.1038/s41586-023-06983-9 PMC11298758

[62] Liang D, Feng Y, Zandkarimi F, Wang H, Zhang Z, Kim J, et al. Ferroptosis surveillance independent of gpx4 and differentially regulated by sex hormones. Cell. 2023;186:2748–64.e22. 37267948 10.1016/j.cell.2023.05.003 PMC10330611

[63] Zheng X, Zhang C. The regulation of ferroptosis by noncoding RNAs. Int J Mol Sci. 24 2023. 10.3390/ijms241713336 PMC10488123 37686142

[64] Xie B, Guo Y. Molecular mechanism of cell ferroptosis and research progress in regulation of ferroptosis by noncoding RNAs in tumor cells. Cell Death Discov. 2021;7:101. 33980834 10.1038/s41420-021-00483-3 PMC8115351

[65] Lee RC, Feinbaum RL, Ambros V. The C. Elegans heterochronic gene Lin-4 encodes small RNAs with antisense complementarity to Lin-14. Cell. 1993;75:843–54. 8252621 10.1016/0092-8674(93)90529-y

[66] Bartel DP. Micrornas: target recognition and regulatory functions. Cell. 2009;136:215–33. 19167326 10.1016/j.cell.2009.01.002 PMC3794896

[67] Iwakawa HO, Tomari Y. The functions of microRNAs: mRNA decay and translational repression. Trends Cell Biol. 2015;25:651–65. 26437588 10.1016/j.tcb.2015.07.011

[68] Pasquinelli AE. MicroRNAs and their targets: recognition, regulation and an emerging reciprocal relationship. Nat Rev Genet. 2012;13:271–82. 22411466 10.1038/nrg3162

[69] Peng Y, Croce CM. The role of MicroRNAs in human cancer. Signal Transduct Target Ther. 2016;1:15004. 29263891 10.1038/sigtrans.2015.4 PMC5661652

[70] Majidinia M, Darband SG, Kaviani M, Nabavi SM, Jahanban-Esfahlan R, Yousefi B. Cross-regulation between notch signaling pathway and miRNA machinery in cancer. DNA Repair (Amst). 2018;66-67:30–41. 29723707 10.1016/j.dnarep.2018.04.002

[71] Fabian MR, Sonenberg N, Filipowicz W. Regulation of mRNA translation and stability by MicroRNAs. Annu Rev Biochem. 2010;79:351–79. 20533884 10.1146/annurev-biochem-060308-103103

[72] Wang J, Zhang L, Jiang W, Zhang R, Zhang B, Silayiding A, et al. Microrna-135a promotes proliferation, migration, invasion and induces chemoresistance of endometrial cancer cells. Eur J Obstet Gynecol Reprod Biol X. 2020;5:100103. 32021975 10.1016/j.eurox.2019.100103 PMC6994408

[73] Chong ZX. Roles of miRNAs in regulating ovarian cancer stemness. Biochim Biophys Acta Rev Cancer. 2024;1879:189191. 39353485 10.1016/j.bbcan.2024.189191

[74] Chen Y, Cheng CS, Chen L. Multifaceted role of microRNA-301a in human cancer: from biomarker potential to therapeutic targeting. Cancer Gene Ther. 2024;31:1754–64. 10.1038/s41417-024-00832-1 39317714

[75] Zhang Y, Guo S, Wang S, Li X, Hou D, Li H, et al. LncRNA Oip5-As1 inhibits ferroptosis in prostate cancer with long-term cadmium exposure through Mir-128-3p/Slc7a11 signaling. Ecotoxicol Environ Saf. 2021;220:112376. 34051661 10.1016/j.ecoenv.2021.112376

[76] Ma LL, Liang L, Zhou D, Wang SW. Tumor suppressor Mir-424-5p abrogates ferroptosis in ovarian cancer through targeting Acsl4. Neoplasma. 2021;68:165–73. 33038905 10.4149/neo_2020_200707N705

[77] Hou Y, Cai S, Yu S, Lin H. Metformin induces ferroptosis by targeting Mir-324-3p/Gpx4 axis in breast cancer. Acta Biochim Biophys Sin (Shanghai). 2021;53:333–41. 33522578 10.1093/abbs/gmaa180

[78] Kapranov P, Cheng J, Dike S, Nix DA, Duttagupta R, Willingham AT, et al. RNA maps reveal new RNA classes and a possible function for pervasive transcription. Science. 2007;316:1484–8. 17510325 10.1126/science.1138341

[79] Yao RW, Wang Y, Chen LL. Cellular functions of long noncoding RNAs. Nat Cell Biol. 2019;21:542–51. 31048766 10.1038/s41556-019-0311-8

[80] Gil N, Ulitsky I. Regulation of gene expression by cis-acting long non-coding RNAs. Nat Rev Genet. 2020;21:102–17. 31729473 10.1038/s41576-019-0184-5

[81] Chen M, Zhang C, Liu W, Du X, Liu X, Xing B. Long noncoding RNA Linc01234 promotes hepatocellular carcinoma progression through orchestrating aspartate metabolic reprogramming. Mol Ther. 2022;30:2354–69. 35192933 10.1016/j.ymthe.2022.02.020 PMC9171153

[82] Bao G, Xu R, Wang X, Ji J, Wang L, Li W, et al. Identification of LncRNA signature associated with pan-cancer prognosis. IEEE J Biomed Health Inf. 2021;25:2317–28. 10.1109/JBHI.2020.3027680 32991297

[83] Kumar D, Gurrapu S, Wang Y, Bae SY, Pandey PR, Chen H, et al. Lncrna Malat1 suppresses pyroptosis and T cell-mediated killing of incipient metastatic cells. Nat Cancer.2024;5:262–82. 38195932 10.1038/s43018-023-00695-9

[84] Zhang B, Bao W, Zhang S, Chen B, Zhou X, Zhao J, et al. Lncrna Hepfal accelerates ferroptosis in hepatocellular carcinoma by regulating Slc7a11 ubiquitination. Cell Death Dis. 2022;13:734. 36008384 10.1038/s41419-022-05173-1 PMC9411508

[85] Lin Z, Song J, Gao Y, Huang S, Dou R, Zhong P, et al. Hypoxia-induced Hif-1α/LncRNA-Pman inhibits ferroptosis by promoting the cytoplasmic translocation of Elavl1 in peritoneal dissemination from gastric cancer. Redox Biol. 2022;52:102312. 35447413 10.1016/j.redox.2022.102312 PMC9043498

[86] Kristensen LS, Andersen MS, Stagsted LVW, Ebbesen KK, Hansen TB, Kjems J. The biogenesis, biology and characterization of circular RNAs. Nat Rev Genet. 2019;20:675–91. 31395983 10.1038/s41576-019-0158-7

[87] Du WW, Zhang C, Yang W, Yong T, Awan FM, Yang BB. Identifying and characterizing CircRNA-protein interaction. Theranostics. 2017;7:4183–91. 29158818 10.7150/thno.21299 PMC5695005

[88] Huang A, Zheng H, Wu Z, Chen M, Huang Y. Circular RNA-protein interactions: functions, mechanisms, and identification. Theranostics. 2020;10:3503–17. 32206104 10.7150/thno.42174 PMC7069073

[89] Margvelani G, Maquera KAA, Welden JR, Rodgers DW, Stamm S. Translation of circular RNAs. Nucleic Acids Res. 2025;53. 10.1093/nar/gkae1167 PMC11724312 39660652

[90] Enuka Y, Lauriola M, Feldman ME, Sas-Chen A, Ulitsky I, Yarden Y. Circular RNAs are long-lived and display only minimal early alterations in response to a growth factor. Nucleic Acids Res. 2016;44:1370–83. 26657629 10.1093/nar/gkv1367 PMC4756822

[91] Hansen TB, Jensen TI, Clausen BH, Bramsen JB, Finsen B, Damgaard CK, et al. Natural RNA circles function as efficient microrna sponges. Nature. 2013;495:384–8. 23446346 10.1038/nature11993

[92] Salmena L, Poliseno L, Tay Y, Kats L, Pandolfi PP. A cerna hypothesis: the Rosetta stone of a hidden RNA language? Cell. 2011;146:353–8. 21802130 10.1016/j.cell.2011.07.014 PMC3235919

[93] Patop IL, Wüst S, Kadener S. Past, present, and future of circRNAs. EMBO J. 2019;38:e100836. 31343080 10.15252/embj.2018100836 PMC6694216

[94] Xu Q, Zhou L, Yang G, Meng F, Wan Y, Wang L, et al. Circil4r facilitates the tumorigenesis and inhibits ferroptosis in hepatocellular carcinoma by regulating the Mir-541-3p/Gpx4 axis. Cell Biol Int. 2020;44:2344–56. 32808701 10.1002/cbin.11444

[95] Jiang Y, Zhao J, Li R, Liu Y, Zhou L, Wang C, et al. Circlrfn5 inhibits the progression of glioblastoma Via Prrx2/Gch1 mediated ferroptosis. J Exp Clin Cancer Res. 2022;41:307. 36266731 10.1186/s13046-022-02518-8 PMC9583503

[96] Kim H, Villareal LB, Liu Z, Haneef M, Falcon DM, Martin DR, et al. Transferrin receptor-mediated iron uptake promotes colon tumorigenesis. Adv Sci (Weinh). 2023;10:e2207693. 36703617 10.1002/advs.202207693 PMC10074045

[97] Liu XS, Yang JW, Zeng J, Chen XQ, Gao Y, Kui XY, et al. Slc2a1 is a diagnostic biomarker involved in immune infiltration of colorectal cancer and associated with M6a modification and CeRNA. Front Cell Dev Biol. 2022;10:853596. 35399515 10.3389/fcell.2022.853596 PMC8987357

[98] Ren Y, Mao X, Xu H, Dang Q, Weng S, Zhang Y, et al. Ferroptosis and Emt: key targets for combating cancer progression and therapy resistance. Cell Mol Life Sci. 2023;80:263. 37598126 10.1007/s00018-023-04907-4 PMC10439860

[99] Wu T, Wan J, Qu X, Xia K, Wang F, Zhang Z, et al. Nodal promotes colorectal cancer survival and metastasis through regulating Scd1-mediated ferroptosis resistance. Cell Death Dis. 2023;14:229. 37002201 10.1038/s41419-023-05756-6 PMC10066180

[100] Miao Q, Deng WQ, Lyu WY, Sun ZT, Fan SR, Qi M, et al. Erianin inhibits the growth and metastasis through autophagy-dependent ferroptosis in Kras(G13d) colorectal cancer. Free Radic Biol Med. 2023;204:301–12. 37217090 10.1016/j.freeradbiomed.2023.05.008

[101] Li H, Liu L, Huang T, Jin M, Zheng Z, Zhang H, et al. Establishment of a novel ferroptosis-related LncRNA pair prognostic model in colon adenocarcinoma. Aging (Albany NY). 2021;13:23072–95. 34610581 10.18632/aging.203599 PMC8544324

[102] Hu M, Yuan L, Zhu J. The dual role of Nrf2 in colorectal cancer: targeting Nrf2 as a potential therapeutic approach. J Inflamm Res. 2024;17:5985–6004. 39247839 10.2147/JIR.S479794 PMC11380863

[103] Xie Y, Zhu S, Song X, Sun X, Fan Y, Liu J, et al. The tumor suppressor P53 limits ferroptosis by blocking Dpp4 activity. Cell Rep. 2017;20:1692–704. 28813679 10.1016/j.celrep.2017.07.055

[104] Ye S, Xu M, Zhu T, Chen J, Shi S, Jiang H, et al. Cytoglobin promotes sensitivity to ferroptosis by regulating P53-Yap1 axis in colon cancer cells. J Cell Mol Med. 2021;25:3300–11. 33611811 10.1111/jcmm.16400 PMC8034452

[105] Lei S, Chen C, Han F, Deng J, Huang D, Qian L, et al. Amer1 deficiency promotes the distant metastasis of colorectal cancer by inhibiting Slc7a11- and Ftl-mediated ferroptosis. Cell Rep. 2023;42:113110. 37682704 10.1016/j.celrep.2023.113110

[106] Li J, Jiang JL, Chen YM, Lu WQ. Klf2 inhibits colorectal cancer progression and metastasis by inducing ferroptosis via the Pi3k/Akt signaling pathway. J Pathol Clin Res. 2023;9:423–35. 37147883 10.1002/cjp2.325 PMC10397377

[107] Zhang C, Liu X, Jin S, Chen Y, Guo R. Ferroptosis in cancer therapy: a novel approach to reversing drug resistance. Mol Cancer. 2022;21:47. 35151318 10.1186/s12943-022-01530-y PMC8840702

[108] Friedmann Angeli JP, Krysko DV, Conrad M. Ferroptosis at the crossroads of cancer-acquired drug resistance and immune evasion. Nat Rev Cancer. 2019;19:405–14. 31101865 10.1038/s41568-019-0149-1

[109] Song M, Huang S, Wu X, Zhao Z, Liu X, Wu C, et al. Ubr5 mediates colorectal cancer chemoresistance by attenuating ferroptosis Via Lys 11 ubiquitin-dependent stabilization of Smad3-Slc7a11 signaling. Redox Biol. 2024;76:103349. 39260061 10.1016/j.redox.2024.103349 PMC11415886

[110] Chen C, Yang Y, Guo Y, He J, Chen Z, Qiu S, et al. Cyp1b1 inhibits ferroptosis and induces Anti-Pd-1 resistance by degrading Acsl4 in colorectal cancer. Cell Death Dis. 2023;14:271. 37059712 10.1038/s41419-023-05803-2 PMC10104818

[111] Shaukat A, Levin TR. Current and future colorectal cancer screening strategies. Nat Rev Gastroenterol Hepatol. 2022;19:521–31. 35505243 10.1038/s41575-022-00612-y PMC9063618

[112] Jain S, Maque J, Galoosian A, Osuna-Garcia A, May FP. Optimal strategies for colorectal cancer screening. Curr Treat Options Oncol. 2022;23:474–93. 35316477 10.1007/s11864-022-00962-4 PMC8989803

[113] Demir H, Beypinar I, Urvay S, Davarcı SE, Baykara M. Prognostic role of pre-operative serum ferritin level in stage 2 colon cancer. Eur Rev Med Pharm Sci. 2021;25:6473–79. 10.26355/eurrev_202111_27091 34787851

[114] Sheng JQ, Li SR, Wu ZT, Xia CH, Wu X, Chen J, et al. Transferrin dipstick as a potential novel test for colon cancer screening: a comparative study with immuno fecal occult blood test. Cancer Epidemiol Biomark Prev. 2009;18:2182–5. 10.1158/1055-9965.EPI-09-0309 19661074

[115] Zhang W, Fang D, Li S, Bao X, Jiang L, Sun X. Construction and validation of a novel ferroptosis-related LncRNA signature to predict prognosis in colorectal cancer patients. Front Genet. 2021;12:709329. 34777458 10.3389/fgene.2021.709329 PMC8581609

[116] Chen W, Deng J, Zhou Y. The construction of a novel ferroptosis-related LncRNA model to predict prognosis in colorectal cancer patients. Med (Baltim). 2023;102:e33114. 10.1097/MD.0000000000033114 PMC9997773 36897681

[117] Shao Y, Jia H, Huang L, Li S, Wang C, Aikemu B, et al. An original ferroptosis-related gene signature effectively predicts the prognosis and clinical status for colorectal cancer patients. Front Oncol. 2021;11:711776. 34249766 10.3389/fonc.2021.711776 PMC8264263

[118] Sato M, Kusumi R, Hamashima S, Kobayashi S, Sasaki S, Komiyama Y, et al. The ferroptosis inducer erastin irreversibly inhibits system X(C)- and synergizes with cisplatin to increase cisplatin’s cytotoxicity in cancer cells. Sci Rep. 2018;8:968. 29343855 10.1038/s41598-018-19213-4 PMC5772355

[119] Chaudhary N, Choudhary BS, Shah SG, Khapare N, Dwivedi N, Gaikwad A, et al. Lipocalin 2 expression promotes tumor progression and therapy resistance by inhibiting ferroptosis in colorectal cancer. Int J Cancer. 2021;149:1495–511. 34146401 10.1002/ijc.33711

[120] Kerkhove L, Geirnaert F, Rifi AL, Law KL, Gutiérrez A, Oudaert I, et al. Repurposing sulfasalazine as a radiosensitizer in hypoxic human colorectal cancer. Cancers (Basel). 2023;15. 10.3390/cancers15082363 PMC10137052 37190291

[121] Li Y, Bi Y, Li W, Piao Y, Piao J, Wang T, et al. Research progress on ferroptosis in colorectal cancer. Front Immunol. 2024;15:1462505. 39359721 10.3389/fimmu.2024.1462505 PMC11444962

[122] Kerkhove L, Geirnaert F, Dufait I, De Ridder M. Ferroptosis: Frenemy of radiotherapy. Int J Mol Sci. 2024;25. 10.3390/ijms25073641 PMC11011408 38612455

[123] Fan F, Liu P, Bao R, Chen J, Zhou M, Mo Z, et al. A dual Pi3k/Hdac inhibitor induces immunogenic ferroptosis to potentiate cancer immune checkpoint therapy. Cancer Res. 2021;81:6233–45. 34711611 10.1158/0008-5472.CAN-21-1547

[124] Benci JL, Johnson LR, Choa R, Xu Y, Qiu J, Zhou Z, et al. Opposing functions of interferon coordinate adaptive and innate immune responses to cancer immune checkpoint blockade. Cell. 2019;178:933–48.e14. 31398344 10.1016/j.cell.2019.07.019 PMC6830508

[125] Li Y, Cheng X. Enhancing colorectal cancer immunotherapy: the pivotal role of ferroptosis in modulating the tumor microenvironment. Int J Mol Sci 2024;25. 10.3390/ijms25179141 PMC11395055 39273090

[126] Zheng H, Liu J, Cheng Q, Zhang Q, Zhang Y, Jiang L, et al. Targeted activation of ferroptosis in colorectal cancer via Lgr4 targeting overcomes acquired drug resistance. Nat Cancer. 2024;5:572–89. 38291304 10.1038/s43018-023-00715-8

[127] Zeng K, Li W, Wang Y, Zhang Z, Zhang L, Zhang W, et al. Inhibition of Cdk1 overcomes oxaliplatin resistance by regulating Acsl4-mediated ferroptosis in colorectal cancer. Adv Sci (Weinh). 2023;10:e2301088. 37428466 10.1002/advs.202301088 PMC10477855

[128] Ou QL, Cheng L, Chang YL, Liu JH, Zhang SF. Jianpi Jiedu decoction reverses 5-fluorouracil resistance in colorectal cancer by suppressing the Xct/Gsh/Gpx4 axis to induce ferroptosis. Heliyon. 2024;10:e27082. 38455561 10.1016/j.heliyon.2024.e27082 PMC10918199

[129] Mu M, Zhang Q, Zhao C, Li X, Chen Z, Sun X, et al. 3-Bromopyruvate overcomes cetuximab resistance in human colorectal cancer cells by inducing autophagy-dependent ferroptosis. Cancer Gene Ther. 2023;30:1414–25. 37558749 10.1038/s41417-023-00648-5 PMC10581902

[130] Balihodzic A, Prinz F, Dengler MA, Calin GA, Jost PJ, Pichler M. Non-coding RNAs and ferroptosis: potential implications for cancer therapy. Cell Death Differ. 2022;29:1094–106. 35422492 10.1038/s41418-022-00998-x PMC9177660

[131] Valashedi MR, Bamshad C, Najafi-Ghalehlou N, Nikoo A, Tomita K, Kuwahara Y, et al. Non-coding RNAs in ferroptotic cancer cell death pathway: meet the new masters. Hum Cell. 2022;35:972–94. 35415781 10.1007/s13577-022-00699-0

[132] Wang D, Tang L, Zhang Y, Ge G, Jiang X, Mo Y, et al. Regulatory pathways and drugs associated with ferroptosis in tumors. Cell Death Dis. 2022;13:544. 35688814 10.1038/s41419-022-04927-1 PMC9187756

[133] Chen D, Fan Z, Rauh M, Buchfelder M, Eyupoglu IY, Savaskan N. Atf4 promotes angiogenesis and neuronal cell death and confers ferroptosis in a Xct-dependent manner. Oncogene. 2017;36:5593–608. 28553953 10.1038/onc.2017.146 PMC5633655

[134] Zhang K, Wu L, Zhang P, Luo M, Du J, Gao T, et al. Mir-9 regulates ferroptosis by targeting glutamic-oxaloacetic transaminase Got1 in melanoma. Mol Carcinog. 2018;57:1566–76. 30035324 10.1002/mc.22878

[135] Martino E, Balestrieri A, Aragona F, Bifulco G, Mele L, Campanile G, et al. Mir-148a-3p promotes colorectal cancer cell ferroptosis by targeting Slc7a11. Cancers (Basel). 2023;15. 10.3390/cancers15174342 PMC10486764 37686618

[136] Elrebehy MA, Abdelghany TM, Elshafey MM, Gomaa MH, Doghish AS. Mir-509-5p promotes colorectal cancer cell ferroptosis by targeting Slc7a11. Pathol Res Pr. 2023;247:154557. 10.1016/j.prp.2023.154557 37229918

[137] Yun ZY, Wu D, Wang X, Huang P, Li N. Mir-214-3p overexpression-triggered chondroitin polymerizing factor (Chpf) inhibition modulates the ferroptosis and metabolism in colon cancer. Kaohsiung J Med Sci. 2024;40:244–54. 38190270 10.1002/kjm2.12802 PMC11895652

[138] Zheng S, Hu L, Song Q, Shan Y, Yin G, Zhu H, et al. Mir-545 promotes colorectal cancer by inhibiting transferring in the non-normal ferroptosis signaling. Aging (Albany NY). 2021;13:26137–47. 34954694 10.18632/aging.203801 PMC8751587

[139] Huang L, Liao C, Xiong Z, Chen Z, Zhang S. Hsa-Mir-526b-5p regulates the sensitivity of colorectal cancer to 5-fluorouracil by targeting Tp53 in organoid models. Biochem Genet. 2025. 10.1007/s10528-025-11045-y. 10.1007/s10528-025-11045-y 39953363

[140] Zhao J, Xu J, Zhang R. Microrna-539 inhibits colorectal cancer progression by directly targeting Sox4. Oncol Lett. 2018;16:2693–700. 30013665 10.3892/ol.2018.8892 PMC6036556

[141] Guo S, Shan S, Wu H, Hao H, Li Z. Recombinant water stress protein 1 (Re-Wsp1) suppresses colon cancer cell growth through the Mir-539/Β-catenin signaling pathway. Mol Biol Rep. 2021;48:7059–65. 34596809 10.1007/s11033-021-06549-w

[142] Yang Y, Lin Z, Han Z, Wu Z, Hua J, Zhong R, et al. Mir-539 activates the Sapk/Jnk signaling pathway to promote ferropotosis in colorectal cancer by directly targeting tipe. Cell Death Discov. 2021;7:272. 34601499 10.1038/s41420-021-00659-x PMC8487425

[143] Liu Y, Liu R, Yang F, Cheng R, Chen X, Cui S, et al. Mir-19a promotes colorectal cancer proliferation and migration by targeting Tia1. Mol Cancer. 2017;16:53. 28257633 10.1186/s12943-017-0625-8 PMC5336638

[144] Chen M, Lin M, Wang X. Overexpression of Mir-19a inhibits colorectal cancer angiogenesis by suppressing Kras expression. Oncol Rep. 2018;39:619–26. 29207158 10.3892/or.2017.6110

[145] Fan H, Ai R, Mu S, Niu X, Guo Z, Liu L. Mir-19a suppresses ferroptosis of colorectal cancer cells by targeting Ireb2. Bioengineered. 2022;13:12021–29. 35599631 10.1080/21655979.2022.2054194 PMC9275930

[146] Liu L, Yao H, Zhou X, Chen J, Chen G, Shi X, et al. Mir-15a-3p regulates ferroptosis via targeting glutathione peroxidase gpx4 in colorectal cancer. Mol Carcinog. 2022;61:301–10. 34727409 10.1002/mc.23367

[147] Li Q, Lei C, Lu C, Wang J, Gao M, Gao W. Linc01232 exerts oncogenic activities in pancreatic adenocarcinoma via regulation of Tm9sf2. Cell Death Dis. 2019;10:698. 31541081 10.1038/s41419-019-1896-3 PMC6754375

[148] Yuan Y, Long Z. Lncrna Linc01232 enhances proliferation, angiogenesis, migration and invasion of colon adenocarcinoma cells by downregulating Mir-181a-5p. J Microbiol Biotechnol. 2023;33:398–409. 36655275 10.4014/jmb.2206.06032 PMC10084753

[149] Zhuang S, Huang Z, Fan H, Wu Z, Liu H. Linc01232 promotes Arntl2 transcriptional activation and inhibits ferroptosis of Crc cells through P300/H3k27ac. Epigenomics. 2024;16:1097–115. 39268727 10.1080/17501911.2024.2387528 PMC11418281

[150] Lan H. Regulatory effects of LncRNA Snhg4 on Mir-25/Fbxw7 axis in papillary thyroid cancer cells. Crit Rev Eukaryot Gene Expr. 2022;32:1–9. 36004691 10.1615/CritRevEukaryotGeneExpr.2022041421

[151] Cao J, Xiao C, Fong CTH, Gong J, Li D, Li X, et al. Expression and regulatory network analysis of function of small nucleolar RNA host gene 4 in hepatocellular carcinoma. J Clin Transl Hepatol. 2022;10:297–307. 35528985 10.14218/JCTH.2020.00175 PMC9039712

[152] Wang F, Quan Q. The long non-coding RNA Snhg4/Microrna-Let-7e/Kdm3a/P21 pathway is involved in the development of non-small cell lung cancer. Mol Ther Oncolytics. 2021;20:634–45. 33816782 10.1016/j.omto.2020.12.010 PMC7995486

[153] Li SQ, Lv F, Xu WT, Yin YX, Wei HT, Li KZ, et al. Lncrna Snhg4 inhibits ferroptosis by orchestrating Mir-150-5p/C-Myb axis in colorectal cancer. Int J Biol Macromol. 2024;268:131961. 38692535 10.1016/j.ijbiomac.2024.131961

[154] Han Y, Gao X, Wu N, Jin Y, Zhou H, Wang W, et al. Long noncoding RNA Linc00239 inhibits ferroptosis in colorectal cancer by binding to Keap1 to stabilize Nrf2. Cell Death Dis. 2022;13:742. 36038548 10.1038/s41419-022-05192-y PMC9424287

[155] Wang XQ, Fan AQ, Hong L. Lncrna Mir210hg promotes the proliferation of colon cancer cells by inhibiting ferroptosis through binding to Pcbp1. Sci Rep. 2025;15:871. 39757305 10.1038/s41598-025-85321-7 PMC11701131

[156] He D, Yue Z, Liu L, Fang X, Chen L, Han H. Long noncoding Rna Abhd11-As1 promote cells proliferation and invasion of colorectal cancer via regulating the Mir-1254-Wnt11 pathway. J Cell Physiol. 2019;234:12070–79. 30537177 10.1002/jcp.27877

[157] Zhang W, Huang X, Shi J. Ezh2-mediated LncRNA Abhd11-As1 promoter regulates the progression of ovarian cancer by targeting Mir-133a-3p. Anticancer Drugs. 2021;32:269–77. 33491971 10.1097/CAD.0000000000001039

[158] Hou S, Zhang X, Yang J. Long non-coding RNA Abhd11-As1 facilitates the progression of cervical cancer by competitively binding to Mir-330-5p and upregulating Mark2. Exp Cell Res. 2022;410:112929. 34793775 10.1016/j.yexcr.2021.112929

[159] Zhuang X, Tong H, Ding Y, Wu L, Cai J, Si Y, et al. Long noncoding RNA Abhd11-As1 functions as a competing endogenous RNA to regulate papillary thyroid cancer progression by Mir-199a-5p/Slc1a5 axis. Cell Death Dis. 2019;10:620. 31409775 10.1038/s41419-019-1850-4 PMC6692390

[160] Bian Y, Xu S, Gao Z, Ding J, Li C, Cui Z, et al. M(6)a modification of LncRNA Abhd11-As1 promotes colorectal cancer progression and inhibits ferroptosis through Trim21/Igf2bp2/ Foxm1 positive feedback loop. Cancer Lett. 2024;596:217004. 38838765 10.1016/j.canlet.2024.217004

[161] Li N, Shen J, Qiao X, Gao Y, Su HB, Zhang S. Long non-coding RNA signatures associated with ferroptosis predict prognosis in colorectal cancer. Int J Gen Med. 2022;15:33–43. 35018112 10.2147/IJGM.S331378 PMC8742603

[162] Wu Z, Zou J, Xie H, Wang J, Huang Y, Liu F, et al. LncRNA Alms1-It1 modulates ferroptosis and immune evasion in colorectal cancer through activating Stat3. J Cell Mol Med. 2024;28:e70103. 39334527 10.1111/jcmm.70103 PMC11436373

[163] Luo Y, Tan W, Jia W, Liu Z, Ye P, Fu Z, et al. The long non-coding RNA Linc01606 contributes to the metastasis and invasion of human gastric cancer and is associated with Wnt/Β-Catenin signaling. Int J Biochem Cell Biol. 2018;103:125–34. 30142387 10.1016/j.biocel.2018.08.012

[164] Luo Y, Huang S, Wei J, Zhou H, Wang W, Yang J, et al. Long noncoding Rna Linc01606 protects colon cancer cells from ferroptotic cell death and promotes stemness by Scd1-Wnt/Β-Catenin-Tfe3 feedback loop signalling. Clin Transl Med. 2022;12:e752. 35485210 10.1002/ctm2.752 PMC9052012

[165] Zou J, Shi X, Wu Z, Zuo S, Tang X, Zhou H, et al. Mrtx1133 attenuates Kras(G12d) mutated-colorectal cancer progression through activating ferroptosis activity Via Mettl14/Linc02159/Foxc2 axis. Transl Oncol. 2025;52:102235. 39657309 10.1016/j.tranon.2024.102235 PMC11683245

[166] Jiang M, Xu L, Lin W, Liu W, Zhang Y, Wang H, et al. LncRNA CRCMSL interferes in phospholipid unsaturation to suppress colorectal cancer progression via reducing membrane fluidity. J Adv Res. 2025. 10.1016/j.jare.2025.02.003.10.1016/j.jare.2025.02.00339921055

[167] Zhang Z, Ye B, Lin Y, Liu W, Deng J, Ji W. Lncrna Otud6b-As1 overexpression promoted gpx4-mediated ferroptosis to suppress radioresistance in colorectal cancer. Clin Transl Oncol. 2023;25:3217–29. 37184781 10.1007/s12094-023-03193-7

[168] Liu R, Wang X, Zhou M, Zhai J, Sun J. Psf-LncRNA interaction as a target for novel targeted anticancer therapies. Biomed Pharmacother. 2024;180:117491. . 10.1016/j.biopha.2024.117491 39332189

[169] Dong Y, He Y, Geng Y, Wei M, Zhou X, Lian J, et al. Autophagy-related LncRNAs and exosomal lncrnas in colorectal cancer: focusing on lncrna-targeted strategies. Cancer Cell Int. 2024;24:328. 39342235 10.1186/s12935-024-03503-1 PMC11439232

[170] Li SQ, Xu WT, Yin YX, Wei HT, Li KZ, Xie MZ, et al. Snhg4-mediated Pten destabilization confers oxaliplatin resistance in colorectal cancer cells by inhibiting ferroptosis. Apoptosis. 2024;29:835–48. 38573492 10.1007/s10495-024-01948-3

[171] Xin Z, Hu C, Zhang C, Liu M, Li J, Sun X, et al. LncRNA-Hmg incites colorectal cancer cells to chemoresistance via repressing P53-mediated ferroptosis. Redox Biol. 2024;77:103362. 39307047 10.1016/j.redox.2024.103362 PMC11447409

[172] Chen H, Zhu P, Zhu D, Jin J, Yang Q, Han X. Role and mechanism of Kiaa1429 in regulating cellular ferroptosis and radioresistance in colorectal cancer. Biomol Biomed. 2024;24:1669–81. 38843497 10.17305/bb.2024.10313 PMC11496857

[173] Yang L, Guan Y, Liu Z. Role of ferroptosis and its non-coding RNA regulation in hepatocellular carcinoma. Front Pharm. 2023;14:1177405. 10.3389/fphar.2023.1177405 PMC10133567 37124203

[174] Lu L, Chen B, Xu Y, Zhang X, Jin L, Qian H, et al. Role of ferroptosis and ferroptosis-related non-coding RNAs in the occurrence and development of gastric cancer. Front Pharm. 2022;13:902302. 10.3389/fphar.2022.902302 PMC9421149 36046827

[175] Xian ZY, Hu B, Wang T, Cai JL, Zeng JY, Zou Q, et al. Circabcb10 silencing inhibits the cell ferroptosis and apoptosis by regulating the Mir-326/Ccl5 axis in rectal cancer. Neoplasma. 2020;67:1063–73. 32567935 10.4149/neo_2020_191024N1084

[176] Li Q, Li K, Guo Q, Yang T. Circrna Circstil inhibits ferroptosis in colorectal cancer Via Mir-431/Slc7a11 axis. Environ Toxicol. 2023;38:981–89. 36840697 10.1002/tox.23670

[177] Fan H, Ding Y, Xiao Z, Li S, Zheng Y. Circ_0075829 regulates ferroptosis and immune escape in colon cancer cells through the Mir-330-5p/Tcf4 Axis. Neoplasma. 2024;71:559–70. 39832206 10.4149/neo_2024_240803N328

[178] Yin W, Xu J, Li C, Dai X, Wu T, Wen J. Circular RNA Circ_0007142 facilitates colorectal cancer progression by modulating Cdc25a expression Via Mir-122-5p. Onco Targets Ther. 2020;13:3689–701. 32431519 10.2147/OTT.S238338 PMC7200250

[179] Wen T, Wu H, Zhang L, Li K, Xiao X, Zhang L, et al. Circular RNA Circ_0007142 regulates cell proliferation, apoptosis, migration and invasion Via Mir-455-5p/Sgk1 axis in colorectal cancer. Anticancer Drugs. 2021;32:22–33. 32889894 10.1097/CAD.0000000000000992

[180] Wang Y, Chen H, Wei X. Circ_0007142 downregulates Mir-874-3p-mediated Gdpd5 on colorectal cancer cells. Eur J Clin Invest. 2021;51:e13541. 33797091 10.1111/eci.13541

[181] Liu X, Zhang J, Nie D, Zeng K, Hu H, Tie J, et al. Comparative transcriptomic analysis to identify the important coding and non-coding RNAs involved in the pathogenesis of Pterygium. Front Genet. 2021;12:646550. 33790949 10.3389/fgene.2021.646550 PMC8005612

[182] Lv W, Liu S, Zhang Q, Hu W, Wu Y, Ren Y. Circular RNA Circcol5a1 sponges the Mir-7-5p/Epac1 axis to promote the progression of keloids through regulating Pi3k/Akt signaling pathway. Front Cell Dev Biol. 2021;9:626027. 33553184 10.3389/fcell.2021.626027 PMC7859531

[183] Wang J, Zhang Z, Zhuang J, Kang D, Song W. Circcol5a1 is involved in proliferation, invasion, and inhibition of ferroptosis of colorectal cancer cells via Mir-1287-5p/Slc7a11. J Biochem Mol Toxicol. 2024;38:e23772. 39030862 10.1002/jbt.23772

[184] Long F, Zhong C, Long Q, Zhu K, Wang J, Yu Y, et al. Circular RNA Rhbdd1 regulates tumorigenicity and ferroptosis in colorectal cancer by mediating the Elavl1/Scd Mrna interaction. Cancer Gene Ther. 2024;31:237–49. 38072968 10.1038/s41417-023-00698-9

[185] Richard F, De Schepper M, Maetens M, Leduc S, Isnaldi E, Geukens T, et al. Comparison of the genomic alterations present in tumor samples from patients with metastatic inflammatory versus non-inflammatory breast cancer reveals Aurka as a potential treatment target. Breast. 2023;69:476–80. 36717329 10.1016/j.breast.2023.01.010 PMC10300569

[186] Zhao Z, Wang H, Kang N, Wang Z, Hou X, Hu L, et al. Aurora kinase a promotes the progression of papillary thyroid carcinoma by activating the Mtorc2-Akt signalling pathway. Cell Biosci. 2022;12:195. 36471438 10.1186/s13578-022-00934-z PMC9721059

[187] Lu K, Yuan X, Zhao L, Wang B, Zhang Y. Comprehensive pan-cancer analysis and the regulatory mechanism of Aurka, a gene associated with prognosis of ferroptosis of adrenal cortical carcinoma in the tumor micro-environment. Front Genet. 2023;13:996180. 10.3389/fgene.2022.996180 PMC9845395 36685952

[188] Hu Y, Wu X, Tan X, Zhang J. Hsa_Circrna_007630 knockdown delays colon cancer progression by modulation of ferroptosis via Mir-506-3p/Aurka axis. J Biochem Mol Toxicol. 2024;38:e23771. 39015057 10.1002/jbt.23771

[189] Huang M, Gao T, Chen X, Yi J, Zhou X. Circ_0087851 suppresses colorectal cancer malignant progression through triggering Mir-593-3p/Bap1-mediated ferroptosis. J Cancer Res Clin Oncol. 2024;150:204. 38642144 10.1007/s00432-024-05643-3 PMC11032280

[190] Yi Q, Feng J, Lan W, Shi H, Sun W, Sun W. Circrna and LncRNA-encoded peptide in diseases, an update review. Mol Cancer. 2024;23:214. 39343883 10.1186/s12943-024-02131-7 PMC11441268

[191] Arshi A, Mahmoudi E, Raeisi F, Dehghan Tezerjani M, Bahramian E, Ahmed Y, et al. Exploring potential roles of long non-coding RNAs in cancer immunotherapy: a comprehensive review. Front Immunol. 2024;15:1446937. 39257589 10.3389/fimmu.2024.1446937 PMC11384988

[192] Lu J, Tan J, Yu X. A prognostic ferroptosis-related LncRNA model associated with immune infiltration in colon cancer. Front Genet. 2022;13:934196. 36118850 10.3389/fgene.2022.934196 PMC9470855

[193] Taniue K, Akimitsu N. The functions and unique features of LncRNAs in cancer development and tumorigenesis. Int J Mol Sci. 2021;22. 10.3390/ijms22020632 PMC7826647 33435206

[194] Ogunwobi OO, Mahmood F, Akingboye A. Biomarkers in colorectal cancer: current research and future prospects. Int J Mol Sci. 2020;21. 10.3390/ijms21155311 PMC7432436 32726923

[195] Hang X, Du Z, Song J. Exploring the prognostic significance of iron death‑related LncRNAs in colorectal cancer: a systematic review and meta‑analysis. Oncol Lett. 2024;27:284. 38736739 10.3892/ol.2024.14417 PMC11083912

[196] Wu Z, Lu Z, Li L, Ma M, Long F, Wu R, et al. Identification and validation of ferroptosis-related LncRNA signatures as a novel prognostic model for colon cancer. Front Immunol. 2022;12:783362. 10.3389/fimmu.2021.783362 PMC8826443 35154072

[197] Cai HJ, Zhuang ZC, Wu Y, Zhang YY, Liu X, Zhuang JF, et al. Development and validation of a ferroptosis-related LncRNAs prognosis signature in colon cancer. Bosn J Basic Med Sci. 2021;21:569–76. 33714257 10.17305/bjbms.2020.5617 PMC8381210

[198] Xu S, Zhou Y, Luo J, Chen S, Xie J, Liu H, et al. Integrated analysis of a ferroptosis-related LncRNA signature for evaluating the prognosis of patients with colorectal cancer. Genes (Basel). 2022;13. 10.3390/genes13061094 PMC9223081 35741856

[199] Guo Y, Wang Z, Tian Y, Li L, Dong J. A ferroptosis-related LncRNAs signature predicts prognosis of colon adenocarcinoma. Life (Basel). 2023;13. 10.3390/life13071557 PMC10381171 37511932

[200] Chen W, Chen Y, Liu L, Wu Y, Fu P, Cao Y, et al. Comprehensive analysis of immune infiltrates of ferroptosis-related long noncoding RNA and prediction of colon cancer patient prognoses. J Immunol Res. 2022;2022:9480628. 35265722 10.1155/2022/9480628 PMC8898846

[201] Chen XQ, Lian K, Chen ZW, Zhang X, Li T, Wu T, et al. Multi-omics characteristics of ferroptosis associated with colon adenocarcinoma typing and survival. Front Biosci (Landmark Ed). 2024;29:13. 10.31083/j.fbl2901013 38287836

[202] Zhang Q, Fan X, Zhang X, Ju S. Ferroptosis in tumors and its relationship to other programmed cell death: role of non-coding RNAs. J Transl Med. 2023;21:514. 37516888 10.1186/s12967-023-04370-6 PMC10387214

[203] Zeng XY, Qiu XZ, Wu JN, Liang SM, Huang JA, Liu SQ. Interaction mechanisms between autophagy and ferroptosis: potential role in colorectal cancer. World J Gastrointest Oncol. 2023;15:1135–48. 37546557 10.4251/wjgo.v15.i7.1135 PMC10401467

[204] Chen J, Qin C, Zhou Y, Chen Y, Mao M, Yang J. Metformin may induce ferroptosis by inhibiting autophagy Via LncRNA H19 in breast cancer. FEBS Open Bio. 2022;12:146–53. 34644456 10.1002/2211-5463.13314 PMC8727937

[205] Liu Z, Wang Q, Wang X, Xu Z, Wei X, Li J. Circular RNA ciars regulates ferroptosis in Hcc cells through interacting with RNA binding protein Alkbh5. Cell Death Discov. 2020;6:72. 32802409 10.1038/s41420-020-00306-x PMC7414223

[206] Liang X, Long L, Guan F, Xu Z, Huang H. Research status and potential applications of Circrnas affecting colorectal cancer by regulating ferroptosis. Life Sci. 2024;352:122870. 38942360 10.1016/j.lfs.2024.122870

[207] Nigita G, Marceca GP, Tomasello L, Distefano R, Calore F, Veneziano D, et al. NCRNA Editing: functional characterization and computational resources. Methods Mol Biol. 2019;1912:133–74. 30635893 10.1007/978-1-4939-8982-9_6

[208] Hosseini K, Ranjbar M, Pirpour Tazehkand A, Asgharian P, Montazersaheb S, Tarhriz V, et al. Evaluation of exosomal non-coding RNAs in cancer using high-throughput sequencing. J Transl Med. 2022;20:30. 35033106 10.1186/s12967-022-03231-y PMC8760667

[209] Kim YJ, Rho WY, Park SM, Jun BH. Optical nanomaterial-based detection of biomarkers in liquid biopsy. J Hematol Oncol. 2024;17:10. 38486294 10.1186/s13045-024-01531-y PMC10938695

[210] Cheng Y, Dong L, Zhang J, Zhao Y, Li Z. Recent advances in microRNA detection. Analyst. 2018;143:1758–74. 29560992 10.1039/C7AN02001E

